# “Where and Whom You Collect Weightings from Matters…” Capturing Wellbeing Priorities Within a Vulnerable Context: A Case Study of Volta Delta, Ghana

**DOI:** 10.1007/s11205-025-03524-x

**Published:** 2025-03-11

**Authors:** Laurence Cannings, Craig W Hutton, Kristine Nilsen, Alessandro Sorichetta

**Affiliations:** 1 School of Geography and Environmental Science, University of Southampton, Southampton, SO17 1BJ UK; 2 Department of Social Statistics and Demography and WorldPop, School of Geography and Environmental Science, University of Southampton, Southampton, SO17 1BJ UK; 3 Dipartimento Di Scienze Della Terra “A. Desio”, Università Degli Studi Di Milano, Via Mangiagalli 34, 20133 Milan, Italy

**Keywords:** Basic needs, Wellbeing, Weighting, Livelihood, Vulnerability

## Abstract

Wellbeing is a crucial policy outcome within sustainable development, yet it can be measured and conceptualised in various ways. Methodological decisions, such as how different components are weighted, can influence wellbeing classification. Many studies utilise equal weighting, assuming each component is equally important; however, does this reflect communities’ lived experiences? This study outlines a multidimensional basic needs deprivation measure constructed from the Deltas, Vulnerability and Climate Change: Migration and Adaptation (DECCMA) survey dataset in Volta Delta, Ghana. Participatory focus groups, interviews and weighting exercises with communities and District Planning Officers (DPOs) explore different subgroups’ wellbeing priorities. Comparative analysis examines the weights provided across genders, decision-making levels and livelihoods; including farming, fishing and peri-urban groups. Objective survey data is also combined with various subjective weights to explore the sensitivity of the overall deprivation rate and its spatial distribution. Significant weight differences are found between livelihoods, with farming and fishing communities weighting “employment”, “bank access”, and “cooperative membership” higher, whereas peri-urban communities apply a greater weight to “healthcare access”. Differences between decision-making levels are also noted. Community members weight “employment” higher, while DPOs assign a larger score to “cooperative membership”. In contrast, consistent weights emerge across genders. Furthermore, applying community livelihood weights produces lower deprivation rates across most communities compared to DPO or equal nested weights. Overall, significant differences between subgroups’ weights and the sensitivity of wellbeing measurement to weighting selection illustrate the importance of not only collecting local weights, but also *where and whom* you collect weightings from matters.

**Supplementary Information:**

The online version contains supplementary material available at 10.1007/s11205-025-03524-x.

## Introduction

Wellbeing is a “favourable state of life desirable for every human being in the world at all times” (Böhnke & Kohler, 2010;5). Research on the measurement and conceptualisation of wellbeing has increased due to its growing importance as a policy outcome (Osei-Tutu et al., 2020), illustrated by all Sustainable Development Goals incorporating elements of wellbeing (UN, 2022). Community wellbeing is a vital prerequisite and outcome of long-term sustainable development within lower-middle-income countries (LMICs) (Helne & Hirvilammi, 2015).

Wellbeing measurement is sensitive to several conceptual and methodological decisions (Cannings et al., 2024), including weighting selection (Becker et al., 1987). Weighting is an “inescapable step” when measuring multidimensional wellbeing (Esposito & Chiappero-Martinetti, 2010), contrasting unidimensional measures such as income poverty which interpret wellbeing from a singular perspective (Voukelatou et al., 2021). The weights applied to different multidimensional components reflect their assumed value to “a good life” (Decancq & Lugo, 2013). “Given the extent of disagreement among reasonable people about the nature of the good life” (Sugden, 1993;1953), wellbeing measurements should account for the diverse values of various groups across different locations (Srinivasan, 1994). As Booysen (2002) recommends, this study incorporates different weightings to identify and support the most vulnerable communities. Weights are collected and compared across communities and District Planning Officers (DPOs) in Volta Delta, Ghana using a novel weighting exercise. These weights are applied to the basic needs deprivation measure, calculated using the Deltas, Vulnerability and Climate Change: Migration and Adaptation (DECCMA) survey.

Basic needs deprivation captures objective wellbeing (OWB) through an “objective-list” approach (Dolan & White, 2007). OWB is defined by universal, tangible components relating to quality of life, such as income or educational attainment (Vaznonienė, 2014), contrasting subjective wellbeing (SWB) which captures individuals’ cognitive judgments and affective reactions to their life and environment (Stone & Mackie, 2013).

Basic needs range from “survival” with access to food/water to “productive survival” with employment, education and political opportunities (Streeten & Burki, 1978). These core human requirements can vary temporally and spatially. For example, agricultural communities may prioritise employment during droughts to ensure food security, whereas urban communities may give precedence to education to increase income generation (Kuepie et al., 2009).

This study has two aims: (i) statistically compare weights between socioecological subgroups, including gender, decision-making level and livelihood (farming/fishing/peri-urban) and (ii) explore the sensitivity of deprivation rates and the spatial distribution to weighting selection. These aims are supported by participatory rural appraisal (PRA) methods (Schreckenberg et al., 2016), including focus groups (FGs) and interviews. These methods were undertaken with DPOs and leadership-selected community members from eight locations to capture further information on actors’ perceptions of a “good life” and potential explanations for different subgroup weightings. Results from the interviews and FGs are presented as verbatim quotes to supplement the discussion of quantitative results. By addressing the research aims, this study makes several novel contributions to the multidimensional wellbeing measurement literature, outlined in Sect. 2.

The mixed method approach unveils significantly different weights across livelihoods and decision-making levels. In particular there is greater emphasis on the role of “employment” in achieving a “good life” amongst farming/fishing communities compared to peri-urban respondents and DPOs. The overall deprivation rate is also sensitive to weighting selection, with community-weighted rates substantially lower than DPO-weighted rates. Therefore, this study highlights where and whom you collect weightings from matters.

This paper firstly places the novel weighting exercise within the broader context of the multidimensional indicator literature. Next, the study area and base dataset are outlined, followed by a description of the PRA and weighting methods. Weights are then statistically compared across socioecological subgroups and applied to all surveyed communities to explore the sensitivity of wellbeing classification to weighting selection.

## Background: weighting methods

Multiple forms of weighting are available when measuring multidimensional wellbeing (Table 1). Equal weighting, used in the UN Human Development Index, ensures consistency across studies. However, this method fails to capture trade-offs and the “hierarchy of needs” across space (Decancq & Lugo, 2013). For example, piped water may be more essential for inland, drought-impacted communities than coastal villages with freshwater aquifers (Wong, 2012). “Nested” weighting gives equal value to each overarching category. However, if the number of indicators differs between categories, the importance given to individual indicators can substantially differ. Nevertheless, if the indicators accurately capture substantial proportions of information about the overarching categories, the number of individual indicators within each category is not a concern (Aguilar & Sumner, 2020; Angulo et al., 2016; Ervin et al., 2018; GSS, 2020).

**Table 1:** Examples of different weighting approaches for multiple deprivation measurement, based on Decancq and Lugo (2013)

Weighting type	Description
Equal	Each measure is weighted equally
Nested	Individual measures are nested within overarching categories. Overarching categories are weighted equally (Alkire & Foster, 2011) Example: 10 measures allocated across 5 categories. Each category has a total weight of 2. Each measure in a category with 4 measures is weighted 0.5
Frequency-based	Deprivations which are less common in the population are weighted higher, whereas widespread deprivations are weighted lower. The weight of the indicator is the inverse of its frequency in the sample Example: 75% households are deprived in food security; a 0.25 weight is applied. 10% households have unsafe sanitation; a weight of 0.90 is applied
Statistical	Principal components analysis (PCA) generates statistical loadings based on the influence or correlation of each indicator to the first principal component; which explains the most variation in overall deprivation. The loadings for the different indicators are applied as weights PCA “creates uncorrelated components, where each component is a linear weighted combination of the initial variables” (Vyas & Kumaranayake, 2006;451)
Community preference-based	Communities rank or give values to different components based on the self-evaluated importance to their overall wellbeing
Expert-based	Scientific experts or policymakers rank different components of multiple deprivation. This may be done via a “budget allocation” exercise

Frequency-based weightings are useful when targeting pockets of deprivation, yet may underestimate basic needs deprivation within LMICs where many have unfulfilled needs. Furthermore, this approach assumes each indicator’s importance is related to the populations’ relative deprivation rather than interpreting them as absolute “ends” (Decancq & Lugo, 2013). Statistical weighting avoids “double counting” by creating uncorrelated linear combinations from correlated indicators. Applying the first component loadings ensures the indicators that correlate the highest with the overall construct are given the most weight, while lowering the importance given to other indicators. However, statistical weights may not reflect real-life importance (Bibi, 2005), while Nardo et al. (2008) also argue that statistical weighting contradicts the purpose of a multidimensional approach, which aims to capture diverse wellbeing elements that may not necessarily be related.

Due to the limitations of these various weighting methodologies and the sensitivity of the basic needs deprivation rate to weighting selection (Cannings et al., 2024), community and expert-preference weights are favoured to mitigate the influence of researchers’ assumptions or arbitrary statistical relationships on wellbeing measurement. This study uses a “nested” approach (Alkire & Foster, 2011) to calculate a baseline measure. This measure is compared to community/expert-preference weighted rates to uncover the potential issues with using external assumptions when applying wellbeing weights. Furthermore, community-preference weights better align with local priorities as “individuals themselves are the best judges of their own situation” (Flik & Praag, 1991;313). Incorporating communities’ perceptions may also restore the “person” into analysis, which could otherwise be lost if viewing wellbeing through detached survey responses (White, 2016). Additionally, by collecting weightings from various subgroups, local challenges can be accounted for and policy buy-in can be improved (Kay & Jost, 2003).

However, “no weighting system is above criticism” (Booysen, 2002;127). For example, due to researcher positionality (Dosu, 2021; Frey & Gallus, 2013), respondents may assume an external researcher in contact with government officials can provide immediate monetary support. Therefore, respondents may emphasise short-term requirements rather than broadly interpreting a “good life” across time. Furthermore, such data is commonly unavailable in surveys, costly to collect, and community members and experts may also hold their own biases (Decancq & Lugo, 2013).

Despite being relatively absent from economic studies, community-preference weights are prevalent within social and health studies (de Kruijk & Rutten, 2007; Kopec & Willison, 2003; Lawson et al., 2013; Pyne et al., 2008; Schaafsma & Gross-Camp, 2021). Applying subjective weights to an OWB measure alters the concept of “wellbeing” (Folwell, 1995), illustrating the capacity for both OWB and SWB elements to be incorporated in sustainable development research (Yang, 2018) to achieve a more comprehensive, locally-grounded understanding of wellbeing (White, 2016).

Despite existing studies capturing community preferences, many apply them homogenously or compare them across large subgroups such as gender (de Kruijk & Rutten, 2007) or country (Abbott et al., 2011; Schaafsma & Gross-Camp, 2021). Studies that average preferences across broad areas may mask local-specific values or challenges. This study is novel in applying different subgroup weightings within a single basic needs deprivation measure. Since budget allocation and certain development decisions are often made at broader scales, an “overall index of disadvantage seems inescapable” (Wolff & De-Shalit, 2007;89). However, ensuring the overall assessment incorporates decomposable elements and local priorities can create opportunities for tailored initiatives, rather than blanket approaches which may entrench existing inequalities.

This novel study captures more granular differences between livelihood groups and decision-making levels. The focus on livelihoods can examine how different environmental conditions, social norms and comparative reference points influence wellbeing priorities (Adger et al., 2002; Ravallion, 2016; Scott, 2006). Other Ghanaian studies have compared basic need preferences between sociodemographic subgroups. However, as is common in approaches incorporating objective and subjective elements (Fleurbaey, 2011; Yang, 2018), a willingness-to-pay approach is often used (Adisah-Atta, 2017; Antwi-Agyei et al., 2021; Korle, 2023). This economic approach arguably fails to distinguish between “potential” and “realised” wellbeing (Gasper, 2007), and may overlook the broader sociocultural controls that influence people’s values.

Collecting DPOs’ perceptions can produce a powerful policy tool. DPOs coordinate and monitor development plans across multiple governmental institutions, such as health, education and employment. The novelty of our approach lies in DPOs being asked to weight the priorities based on what they perceive to be most important to their communities, rather than themselves. Therefore, by identifying differences between district-level perspectives and livelihood groups’ needs, this study can highlight the benefit of heterogenous local-scale wellbeing initiatives (Kim et al., 2015). Furthermore, disconnects between DPOs and local communities’ perceived values can emphasise the need for greater capacity within local government to create platforms for cross-scale dialogue. These mechanisms could help reduce any discrepancies that may exist between local government priorities and community needs, and improve the local relevance of development initiatives.

This study advances the preference weighting approach by comparing basic needs priorities between local communities with different livelihoods and landscapes, and local decision-makers. Existing studies capture different policymakers’ preferences in Ghana regarding health and wellbeing interventions (Baltussen et al., 2006; Jehu-Appiah et al., 2008); however, these priorities are not compared to local community perspectives. These studies used Multi-Criteria Analysis, a common approach for capturing various stakeholders’ rankings and opinions on desired interventions and policy priorities (Gebre et al., 2021). This study differs from these approaches in that it does not include the ranking of policy strategies. Instead, it includes a process to capture local values when measuring multidimensional wellbeing outcomes, which can support the design of initiatives aimed at improving local communities’ lived experiences.

Previous research has explored the impact of different weighting approaches on rates of multidimensional poverty or deprivation. For example, Libório et al. (2022) illustrated that a composite index of social vulnerability in Brazil was comparable across equal weighting, data-driven, and expert opinion approaches; however, the weights assigned to individual indicators varied substantially. Similarly, de Kruijk and Rutten (2007) observed a similar rate of multidimensional poverty in the Maldives when using a bespoke index with community preference weights and the UNDP Human Development Index which applies equal weighting. In contrast, Datt (2019) found that multidimensional poverty in the Philippines decreased significantly more when using subjective weights collected from a social survey compared to nested or frequency-based weights (2004–2013). The inconsistency in the results across the literature underscores the need to examine the influence of different weighting approaches within the local context. Moreover, existing studies primarily focus on comparisons to equal weighting baselines. This study compares community and local government preference weights to a “nested” baseline, while also undertaking a novel approach by comparing the preferences of different community subgroups.

Furthermore, Wolff and De-Shalit (2007) state wellbeing policy should be implemented to prevent clusters of disadvantage. This study captures different groups’ priorities and applies them to secondary survey data to explore the impact of different weightings on the spatial distribution of basic needs deprivation. While existing studies have examined the influence of different weighting approaches on the spatial properties of multidimensional deprivation (Correa Machado et al., 2023; Libório et al., 2022, 2024), much of the research focuses on comparing data-driven techniques and expert opinions (Wehbe & Baroud, 2024). To our knowledge, no studies have explored how the weights of different subgroups, including livelihood type, affect the spatial distribution of multidimensional wellbeing within an LMIC context. This approach can support policy in targeting locations where both the value attached to certain basic needs and the level of deprivation are high.

Utilising community weights acknowledges how wellbeing is “relational” (White, 2016), meaning it is formed and reproduced within a specific temporal and spatial context. Therefore, wellbeing research should be context-specific to uncover local nuances behind broader patterns. This study focuses on the case study site of Volta Delta, Ghana.

## Data and Methodology

### Study Area

Deltas, many of which are located in LMICs, are often targeted by international development (Foufoula-Georgiou et al., 2013; Szabo et al., 2016). This is due to the combination of high population densities (Ericson et al., 2006), economic potential, environmental vulnerability, and high dependency upon ecosystem services for human and material wellbeing (Kuenzer & Renaud, 2012).

Despite being set in an environmentally vulnerable context, this paper does not focus explicitly on communities’ environmental experiences. Conversely, it focuses on the weighting exercises undertaken during community FGs and DPO interviews, implicitly addressing environmental themes by comparing priorities across landscapes and livelihoods.

Volta Delta was selected due to its diverse livelihoods, landscapes, and economic, environmental and political challenges. These varied characteristics provided a space to research different socioecological subgroups’ experiences, values and wellbeing priorities.

**Fig.1:**
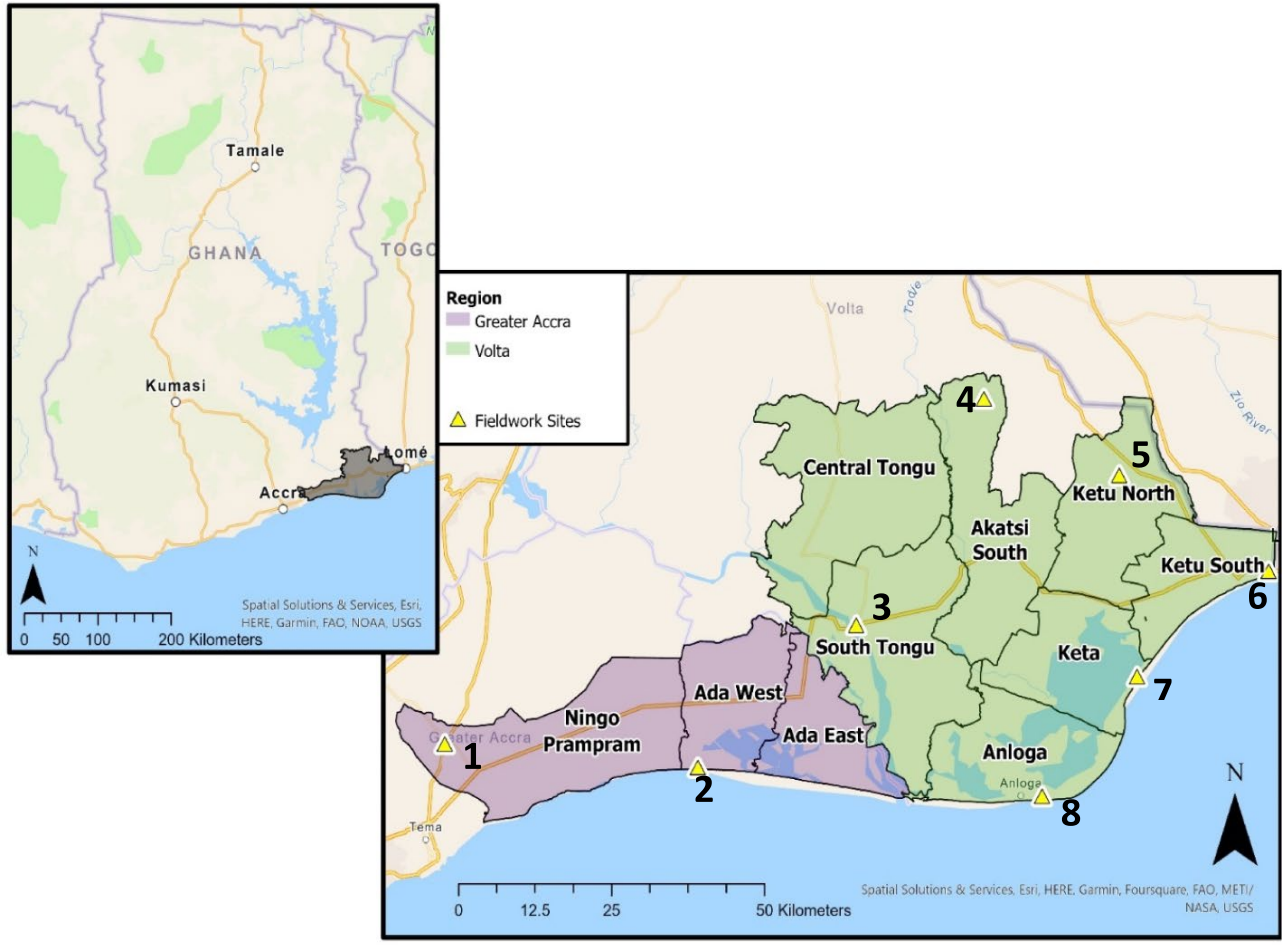
Map of Volta Delta, with regional (Volta and Greater Accra) and district boundaries. The eight selected communities where PRA methods were undertaken, situated within eight different districts, are also illustrated. *Note, the GPS coordinates, which were offset to ensure confidentiality, illustrate Nyitawuta (Site 4) to be within the Akatsi South district; however, upon arrival we were informed it falls under the jurisdiction of Akatsi North district to the east*

Volta Delta is located across Volta and Greater Accra regions (Fig. 1), containing 4% of the national population (945,827) (Adjei et al., 2019). The landscape is characterised by the Volta River, Songor Lagoon, Keta Lagoon, and various landcover types, including cropland, grassland, wetland, and built-up areas (Jayson-Quashigah, 2016). Primary sector livelihoods, involving natural resource extraction, contribute most to delta GDP (29%), with 22% produced by agriculture and 7% by fishing. Trade, transport and industry (inc. salt mining and food processing) contribute 20% GDP each, and construction 11% (Cazcarro et al., 2018). Approximately one-third of individuals work within agriculture, higher than the proportion of GDP generated. This disparity is driven by high levels of subsistence farming, and low productivity and technology access (Arto et al., 2020).

Volta Delta contains two main ethnic groups; Ewe and Ga-Dangme. Both groups are patrilineal, meaning assets and inheritance rights are passed along male bloodlines. This results in gendered imbalances in asset ownership and human capital, such as education (Codjoe et al., 2020; Kutsoati & Morck, 2014). For example, educational attainment is low, with 30% of 15 + year-olds being illiterate, yet literacy levels are higher amongst males (GSS, 2012). Low attainment persists despite state-led “Free Compulsory Universal Basic Education” (FCUBE) being launched in 1995 (Palmer, 2005).

GSS (2020) Living Standards Survey (2011–2018) also illustrated regional variation in multidimensional OWB, with 58.2% deprived in Volta and 22.5% in Greater Accra. Volta is one of four regions with a deprivation rate higher than the national average, driven by low health insurance, poor nutrition, unsafe latrines, and low school attendance/attainment. In contrast, Greater Accra has the lowest regional rate, with higher proportions of adequate housing and clean water, improved material assets, and electricity/cooking fuel access (GSS, 2020). This regional disparity stems from (post)colonial investment patterns (Jedwab & Moradi, 2016), with Greater Accra historically favoured due to its abundant natural resources and international port (Kambala, 2022). See Appendix A for more information on regional differences in human, physical and social resources (Awanyo & Attua, 2018).

### Secondary Dataset

The DECCMA household survey in Volta Delta primarily aimed to explore the effectiveness of adaptation and migration within the context of changing climatic conditions. This study alternatively uses this secondary data to construct the basic needs deprivation measure and its multidimensional components. These indicators were weighted by the scores provided during community FGs and DPO interviews (Sect. 3.5).

The survey was undertaken with household heads using face-to-face interviews (April–June 2016). A two-stage clustered sampling strategy aimed to survey 1500 households. Households were stratified into five strata based on environmental risk. Fifty enumeration areas, classified during the 2010 Census, were randomly selected proportional-to-size from the strata. Thirty occupied residential dwellings from each enumeration area were randomly selected. A 91% response rate was achieved (1364 households).^1^ Further information on the survey strategy is available in Atiglo et al. (2022), where the secondary data was used to investigate coastal vulnerability.

The survey collected various data, including households’ finances, self-reported climate risks, sociodemographic characteristics, and assets. Enumeration areas’ GPS coordinates also enabled the construction of location-based deprivation indicators, such as “healthcare access” (Appendix B).

Descriptive sociodemographic statistics are presented in Appendix C to provide a profile of the DECCMA sample. Households were located in Volta (64%) and Greater Accra (36%) regions. Most household heads were male (59%), had lived in the community their entire life (54%), and received no/primary education (57%).

### Methodology

#### Site Selection

Eight communities included within the DECCMA survey were selected for qualitative fieldwork (Fig. 1). These locations were selected based on key themes identified in the survey data, including areas with distinct wellbeing outcomes, livelihoods and landscape characteristics. For example, Nyitawuta (Site 4) was selected due to high grassland coverage, remoteness from urban towns, and higher proportions of subjectively happy households experiencing expenditure poverty. Sogakope (Site 3) was selected due to its non-primary livelihoods, such as tourism, proximity to the river, built-up landscapes, and a higher proportion of subjectively unhappy households not experiencing poverty. See Appendix D for further justification of the site selection.

Permission to enter communities was received from the Chief Executive, Chief Planning Officer or DPO from each of the eight districts. Upon arrival, participant information sheets and an approval letter from the University of Ghana were provided to all participants and local leaders. Liaising with local gatekeepers was necessary to ensure acceptance within local communities (Maunganidze, 2019) and that culturally-valued norms were followed (Mfoafo-M’Carthy & Grischow, 2022).

Qualitative fieldwork was conducted February–March 2023, approximately seven years after DECCMA. Therefore, contextual changes, such as the national economic downturn (IMF, 2023; World Bank, 2022), must be acknowledged when interpreting communities' wellbeing.

#### Focus Groups

Two semi-structured FGs were conducted at each site: one female and one male. 6–10 participants were targeted for each FG (Table 2). Female and male research assistants (RAs) led their corresponding FGs to ensure participants were comfortable disclosing information (Yager et al., 2013). Due to the social structure in Ghana (Codjoe et al., 2020), FGs were split by gender to reduce social desirability bias. For example, women may be unable to speak about the negative wellbeing effects of male actions (Tsekleves et al., 2020). However, power dynamics along other social lines, such as political influence or age, could still have generated bias (Farr, 2018). See Appendix E for additional FG information, including respondents’ age profiles.

**Table 2:** Number of focus group and semi-structured interview participants by gender

		Focus groups		Interviews	
Location	Livelihood group	No. male participants	No. female participants	No. community participants	No. DPO participants
1) Afienya	Peri-urban	7	6	1M	1F
2) Anyamam	Fishing	8	8	1M, 1F	1F
3) Sogakope	Peri-urban	6	10	1M, 1F	1M
4) Nyitawuta	Farming	8	8	1M, 1F	1M
5) Awlikope	Farming	8	8	1M, 1F	1M
6) Aflao	Fishing	7	8	1M, 1F	1M
7) Kedzi	Fishing	9	7	2M	1M
8) Anloga	Farming	10	8	1M, 1F	1M

The “peri-urban” livelihood group incorporates non-primary occupations which do not involve raw material extraction; for example, hospitality, construction, or transportation. See Sect. (3.5.2) for further information on community livelihood group classification

All participants provided written consent. RAs translated the information if necessary. All FGs were undertaken in Ewe or Dangbe, except the male FG in Afienya (Site 1), where discussions were in English. Therefore, translations may have altered the meaning of certain questions/responses. For example, “wellbeing” is often interpreted to specifically mean “good health” or “good character” (Osei-Tutu et al., 2020). Therefore, following discussions with RAs, and engaging with literature (Dzokoto et al., 2019), respondents were alternatively asked about their “good life”.

FGs bridge scientific research and local knowledge (Cornwall & Jewkes, 1995) by efficiently gathering information (Henningsen et al., 2020) on different groups’ experiences and wellbeing conceptualisations (Flowerdew & Martin, 2013). FG discussions were centred around the weighting exercise (Sect. 3.5). Communities discussed how environmental conditions, governance structures and other contextual factors impact, and are impacted by, access to basic needs.

A semi-structured approach creates a more relaxed environment, where the researcher becomes a “moderator” rather than an “investigator” (O.Nyumba et al., 2018). This approach can stimulate higher idea generation (Coenen et al., 2012;367), increase participant disclosure (Guest et al., 2017), and empower marginalised groups (Peek & Fothergill, 2009; Wilkinson, 1998).

FG respondents were primarily selected by assemblymen and committee members in each community (Tsekleves et al., 2020). Despite discussions with local gatekeepers regarding the importance of accessing diverse participants, it was acknowledged that access to certain voices may have been restricted. This limitation was observed in Nyitawuta (Site 4), where despite observing more deprived areas in the highly remote community, a proportion of the selected male FG respondents had experience travelling to Accra. However, to ensure positive relationships were maintained, control over participant selection had to be surrendered to an extent (Dosu, 2021).

#### Semi-Structured Interviews

Two semi-structured interviews were conducted at each site (Table 2) with individuals encountered during FGs or community walks.^2^ Individuals were selected based on the interest in their FG inputs and livelihoods, particularly if similar livelihoods were not represented in FGs. Each site targeted one female and one male interview.^3^ All community interviewees provided written consent. All FG and interview discussions were audio-recorded and transcribed.

Semi-structured interviews, which incorporate pre-determined discussion topics yet allow “flexibility for participants to bring their own personality and perspective” (Barrett & Twycross, 2018;63), were essential in allowing participants to freely define their wellbeing, rather than being restricted by researchers’ assumptions. Discussions aimed to understand individuals’ financial, social and environmental challenges, and wellbeing conceptualisations. Interviewees were also asked about their comparative reference points, which are key factors influencing SWB outcomes (Kangmennaang et al., 2019). Interviews were led by the same gendered RA, or the research team if the participant was comfortable conversing in English (Appendix E). Nevertheless, RAs were present at all interviews to support any necessary translations.

Eight DPOs were also interviewed face-to-face within governmental offices or via telephone. Spoken consent was received during telephone interviews. These interviews were flexible, drawing on key themes from community FGs. DPO interviews enabled broader discussions regarding district-level challenges and policy decisions. A similar weighting exercise (Sect. 3.5) was also carried out with DPOs to compare their viewpoints on the community’s priorities with those of the FG respondents.^4^

This paper follows a sequential mixed method approach, where qualitative data in the form of verbatim quotes^5^ from interviews and FGs is presented in the “Discussion” (Sect. 5) to aid the interpretation of quantitative weighting data and enrich findings with locally-grounded perceptions. The aim was not to quantify thematic codes, but to extract key ideas across the study sites to evaluate statistical results.

### Basic Needs Deprivation

This study focuses on the influence of different actors’ wellbeing priorities upon the objective, basic needs deprivation measurement. Before exploring different weightings, a baseline comparative measure was calculated.

The baseline basic needs deprivation measure was captured using Alkire and Foster’s (2011) “dual cut-off” count method. Twelve basic needs indicators (Table 3), adapted from existing basic needs studies (GSS, 2020; Santos & Villatoro, 2018; Streeten, 1984), were constructed from the DECCMA dataset. The low correlation amongst most indicators^6^ suggests the measures capture different information (Appendix F). A “deprivation” threshold was applied for each indicator. For example, a household was deprived in “education” if all members aged 15 + had not completed a basic education. Another example is the “healthcare access” indicator, which due to the common absence of objective health data within social surveys, serves as a proxy for a component of “human capital”.^7^ This measurement is a frequently used methodology, where distance is assumed to capture information on healthcare access and health outcomes (Aboaba et al., 2023; Dotse‑Gborgbortsi et al., 2020, 2022; Simeos & Almeida, 2014; Titus et al., 2015). It is acknowledged that using “distance” to capture human capital could be a limitation, as hospital proximity does not necessarily reflect health status (Kelly et al., 2016). However, the importance of “good health” for a “good life” in Ghana (Osei-Tutu et al., 2020), meant it was essential to include an element of “health” within the basic needs measure. See Appendix B for further information on the deprivation indicators’ thresholds.

**Table 3:** Individua﻿l deprivation indicators incorporated within the basic needs deprivation measure, grouped within the Sustainable Livelihoods Framework capitals (Scoones, 1998); produced using DECCMA data. “Nested” baseline weights are also presented

Capital group	Basic need	Indicator	“Nested” baseline weight
Financial	Employment	One or more household members are unemployed	1
	Excess capital	Monthly expenditure on food > 60% total expenditure	1
	Bank access	No access to bank or loan service	1
Human	Education	All household members aged 15 + without basic education	1.5
	Healthcare access	> 5km from nearest hospital	1.5
Social	Cooperative membership	Not a member of a community cooperative network	1.5
	Network size	Under 3 family/friends with migration experience	1.5
Physical	Roof quality	Low-quality roof material	0.6
	Latrine	Unsafe latrine facility	0.6
	Drinking water	Unsafe drinking water source	0.6
	No overcrowding	Overcrowded household	0.6
	Homeownership	Home is not owned	0.6

Following Alkire and Santos’ (2010) approach, “nested” weighting was applied to the indicators to create a “baseline” measure. Indicators were grouped by financial, human, social and physical capitals, as outlined in the Sustainable Livelihoods Framework (SLF) (Scoones, 1998), with each group weighted equally overall (Table 3). Drawing on the SLF’s objective types of capital ensured the different resources that control livelihood and adaptive strategies (Burney & Naylor, 2012; Forkuo et al., 2021), and are depended upon to “durably sustain people’s basic needs” (Gaillard et al., 2009;120), were considered within the multidimensional measure. Using broad capital categories to structure the basic needs measure also provided flexibility, enabling the inclusion of various indicators available within the dataset.

As the survey dataset was designed primarily to examine migratory behaviour, proxy indicators were used (Appendix B), guided by existing multidimensional deprivation studies. The indicators do not capture the SLF’s capital categories in their totality; however, within the dataset restraints, they were designed to capture key documented requirements within an LMIC context.

Next, a second threshold defining the proportion of deprivations needed to be deprived “overall” was applied. A household was defined as deprived “overall” if it experienced at least 50% weighted deprivations, meaning the cumulative nested weight was equal to, or greater than, 6 (González et al., 2021; Hjelm et al., 2016a, 2016b). The 50% threshold was selected as it provided the most comparable result to GSS (2020) multiple deprivation estimates. It also fulfilled existing criteria for multiple deprivation classification, including the equivalent of two full “nested” deprivations, used in UNICEF Child Poverty reports (Alkire & Foster, 2011), and the requirement for the majority of deprivation components to be experienced (Aguilar & Sumner, 2020).

### Weighting exercise

This section outlines methodologies for collecting, comparing, calculating and applying weights to the basic needs deprivation measure.

#### Collection

The weighting exercise was undertaken at each community FG and DPO interview. The basic needs deprivation measure was selected as it consists of decomposable components which could be individually scored (Alkire & Foster, 2011).

**Fig. 2:**
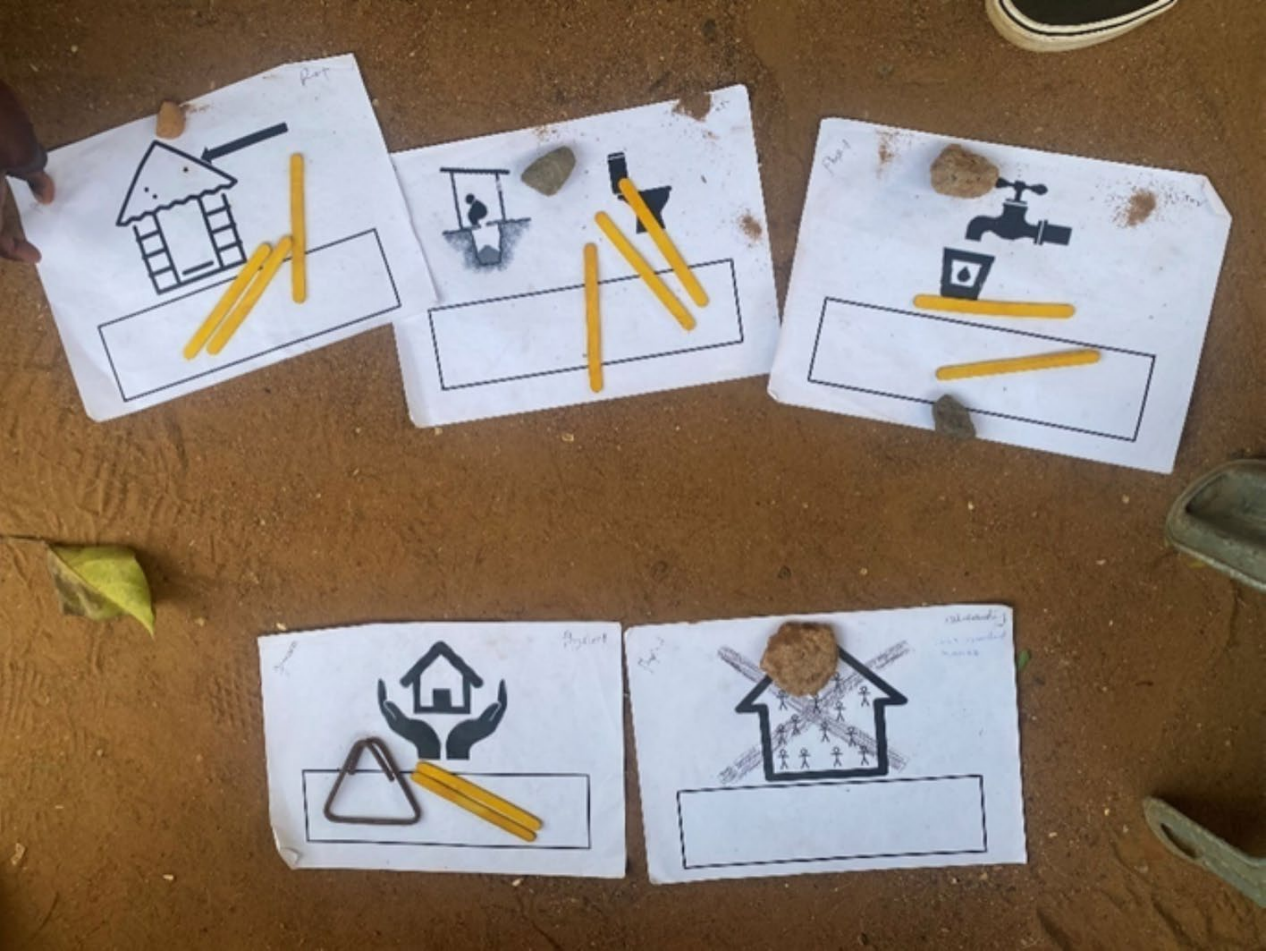
Photograph of the weighting scores distributed across the physical capital components during a community focus group

A direct weighting approach was used, where participants distributed ten sticks across the basic needs components depending on the importance to their “good life” (Shaffer, 2013) (Fig. 2). DPOs weighted components based on their perception of their communities’ priorities. To minimise cognitive load, participants distributed weights within one capital group at a time and then across the four overarching groups (Table 4). Pictograms supported all components to ease interpretation and facilitate participation from illiterate individuals (Schaafsma & Gross-Camp, 2021) (Appendix G).

**Table 4:** The different rounds of weighting undertaken during community focus groups and district planning officer (DPO) interviews

Round 1: Financial capital—>	Employment/ livelihood	Excess capital (beyond food expenditure)	Bank access (formal or informal)	x	x
Round 2: Human capital—>	Education	Healthcare access	x	x	x
Round 3: Social capital—>	Cooperative membership	Family/friends network	x	x	x
Round 4: Physical capital—>	Roof quality	Safe drinking water	Safe latrine facilities	No overcrowding in the home	Home ownership
Round 5: Overall capitals—>	Financial capital	Human capital	Social capital	Physical capital	

A “weighting” rather than “ranking” approach was favoured to allow for unequal differences between components, and to acknowledge the “hierarchy of needs” (Sulemana, 2016). The weighting approach allows participants to weight certain components equally and disregard them if they were unnecessary for a “good life”. It is recognised that the equal distribution of the ten sticks was not possible in Rounds 1 & 5 (Table 4). However, viewing results from Round 5 as an example, 64% of community respondents gave a weight of 0 or 1 to at least one capital, suggesting most respondents did not aim to distribute weights equally across the four groups.

Each individual distributed their sticks in turn, rather than deciding upon a score collectively, to avoid certain individuals projecting their views upon the group (Flowerdew & Martin, 2013). Local leaders were excluded from FGs to minimise their influence upon responses. However, due to time and space restraints, each individual distributed their weights in front of the group, which could have induced social desirability bias. Future research should anonymise the process.

Nevertheless, score mimicking was not a major issue. Of the 126 community FG participants, 110 did not allocate their sticks first. These individuals completed 550 weighting rounds (110 participants × 5 rounds). Only 15% of these rounds matched the scores of the corresponding first respondent. See Appendix H for individual-level scores.

#### Comparative Analysis

Individuals’ scores were used for comparative analysis. Scores for individual indicators were adjusted by the “overall capital” score (Table 4 [Round 5]). For example, if “employment” was given 3/10 points in Round 1, and “financial capital” was scored 5/10 in Round 5, the individual’s “employment” score would be 1.5 (3 *[score given to “employment” in Round 1]* $\times$ 0.5 *[proportion of points given to “financial capital” in Round 5]*).

Mann–Whitney and Kruskal–Wallis^8^ tests were undertaken to determine whether mean ranked scores for different components differed by community subgroup: livelihood, gender and decision-making level (Table 5). These non-parametric tests have been used in previous studies comparing scores/ranks from participatory methods (Ahmad Yahaya et al., 2022; Ahmed & Jena, 2023; Bassachs et al., 2020; Dicker et al., 2019; Jones et al., 2019; Koko et al., 2020; Nthiwa et al., 2019; Schaafsma & Gross-Camp, 2021) as they do not require normal distributions, and perform well with small sample sizes and unequal subgroups (Table 5). Following significant Kruskal–Wallis results, stochastic dominance tests detected which pairwise livelihood groups’ scores significantly differed. Note, due to the study sites being purposively selected, the results are not generalizable across the population.

**Table 5:** Sample sizes for the different socioecological subgroups included within comparative analysis

Subgroup	No. participants
*Gender*	
Male	63
Female	63
*Livelihood*	
Farming	50
Fishing	47
Peri-urban	29
*Decision-making level*	
Community	126
District Planning Officer (DPO)	8

Most visited communities possessed multiple livelihood types. This analysis defined the dominant livelihood type through observations. For example, Anloga’s (Site 8) respondents mentioned farming and fishing livelihoods; however, irrigated farmland was most visible in the immediate community. Three livelihood groups were selected (Table 5). It is acknowledged that communities are not homogeneous; however, limited resources and control over participant selection restricted the ability to capture the multifaceted social, political and economic intracommunity hierarchies.

To compare communities’ perceptions to objective circumstances and hypothesise why individuals prioritised certain basic needs, a similar Kruskal–Wallis analysis was undertaken between livelihoods to explore differences in the proportion of households experiencing the 12 objective basic need deprivations. This analysis, using DECCMA data, is interpreted alongside weighting comparisons.

#### Weight Calculation

Individuals’ scores were summed by subgroup (livelihood, gender, decision-making level) and converted into weights for each basic needs deprivation indicator (Eq. 1). For instance, to create the “employment” weight for the “farming” livelihood group, the proportion of total points given to “employment” by all farming participants in Round 1 (relative to “excess capital” and “bank access”) was multiplied by the proportion of total points given to “financial capital” by all farming participants in Round 5 (relative to “human”, “social” and “physical” capitals) (Table 4). This value was then multiplied by 12 to ensure that all components' weights sum to 12, maintaining comparability with the baseline 'nested' approach (Alkire & Foster, 2011). Radar charts were produced to visually compare different subgroups’ weightings to baseline weights.

1 $$Basic\; needs\; component\; subgroup\; weighting\; =\;\left(\left({x}_{i}/\sum {x}_{i}\right) \times \left({y}_{j}/\sum {y}_{j}\right)\right) \times 12$$

Equation 1 Methodology for transforming community-preference scores into basic needs deprivation weights. All weights total 12 to ensure comparability with the nested “baseline”. Where ${\text{x}}_{\text{i}}$ represents the individual component (x) [Rounds 1–4] within the capital group (i), and ${\text{y}}_{\text{j}}$ represents the corresponding capital weighting (y) [Round 5] as a proportion of the four capitals (j).

#### Applying Weights

The calculated subgroup weights were applied to the 12 binary indicators incorporated within the basic needs deprivation measure (Table 3). Two alternative “overall” deprivation rates were calculated and compared to the baseline rate by applying subgroup weights to all 50 DECCMA communities: (i) “DPO rate”; applied summed weights from all 8 DPOs, (ii) “community rate”; applied weights from the livelihood group most relevant to each community. The different livelihood group weights were also applied to all households to examine how applying one subgroup’s weights universally across the DECCMA sample impacted the deprivation rate.

Each surveyed community’s livelihood type was determined by the proportion of households with at least one crop farmer, fisher, and salaried employee/business owner (“peri-urban”) within the DECCMA dataset. If this method was inconclusive, landscape characteristics, including the proportion of crop/grassland (“farming”) and built-up (“peri-urban”) landcover within a 2km community buffer^9^ and the proximity to coast/inland water (“fishing”), were used to apply the most relevant typology. Applying different subgroups’ weightings within a single measure can support the identification of target areas where the level of deprivation and the perceived importance of certain basic needs are highest.

It is acknowledged that this method has several limitations. Firstly, communities’ livelihoods may have altered since the DECCMA survey. Secondly, many communities contained mixed livelihoods, so applying a single set of weights may not be relevant to all. Furthermore, community-preference weights were applied to a pre-determined list of basic needs, mirroring criticisms of paternalistic “objective-list” methods (Adato & Meinzen-Dick, 2002; Agrawal, 2008; Dolan & White, 2007). This limitation was accepted due to the weights being applied to secondary data. However, due to these limitations, future fieldwork should revisit all 50 sampled locations to more accurately classify livelihood typologies and incorporate preliminary work to capture communities’ self-defined priorities (Schaafsma & Gross-Camp, 2021).

Maps are also produced to illustrate differences in the proportion of households defined as “deprived” within each sampled community when applying community livelihood weights, compared to “nested” and DPO weights. Examining spatial differences in basic needs deprivation when applying different weights can support policy by detecting deprived areas that may have been overlooked if alternative weights were used.

## Results

This section outlines the weighting results from the community FGs and DPO interviews. The basic needs deprivation measure contains 12 individual indicators. The most prevalent deprivation in the DECCMA sample is “healthcare access” (82%), while the least frequent is overcrowding (16%) (Table 6). Weighting scores for each indicator are statistically compared across livelihood, gender, and decision-making subgroups. See Appendix H for individual and community-level scores.

**Table 6:** Number of households (and percentage) classified as deprived in the 12 basic needs indicators; produced using DECCMA data

Capital group	Basic need	Indicator	Deprived household count (%)
Financial	Employment	One or more household members are unemployed	328 (24%)
	Excess capital	Monthly expenditure on food > 60% total expenditure	776 (57%)
	Bank access	No access to bank or loan service	823 (60%)
Human	Education	All household members aged 15 + without basic education	453 (33%)
	Healthcare access	> 5km from nearest hospital	1120 (82%)
Social	Cooperative membership	Not a member of a community cooperative network	1167 (86%)
	Network size	Under 3 family/friends with migration experience	471 (35%)
Physical	Roof quality	Low-quality roof material	738 (54%)
	Latrine	Unsafe latrine facility	557 (41%)
	Drinking water	Unsafe drinking water source	304 (22%)
	No overcrowding	Overcrowded household	222 (16%)
	Homeownership	Home is not owned	195 (14%)

### Livelihood

**Fig.3:**
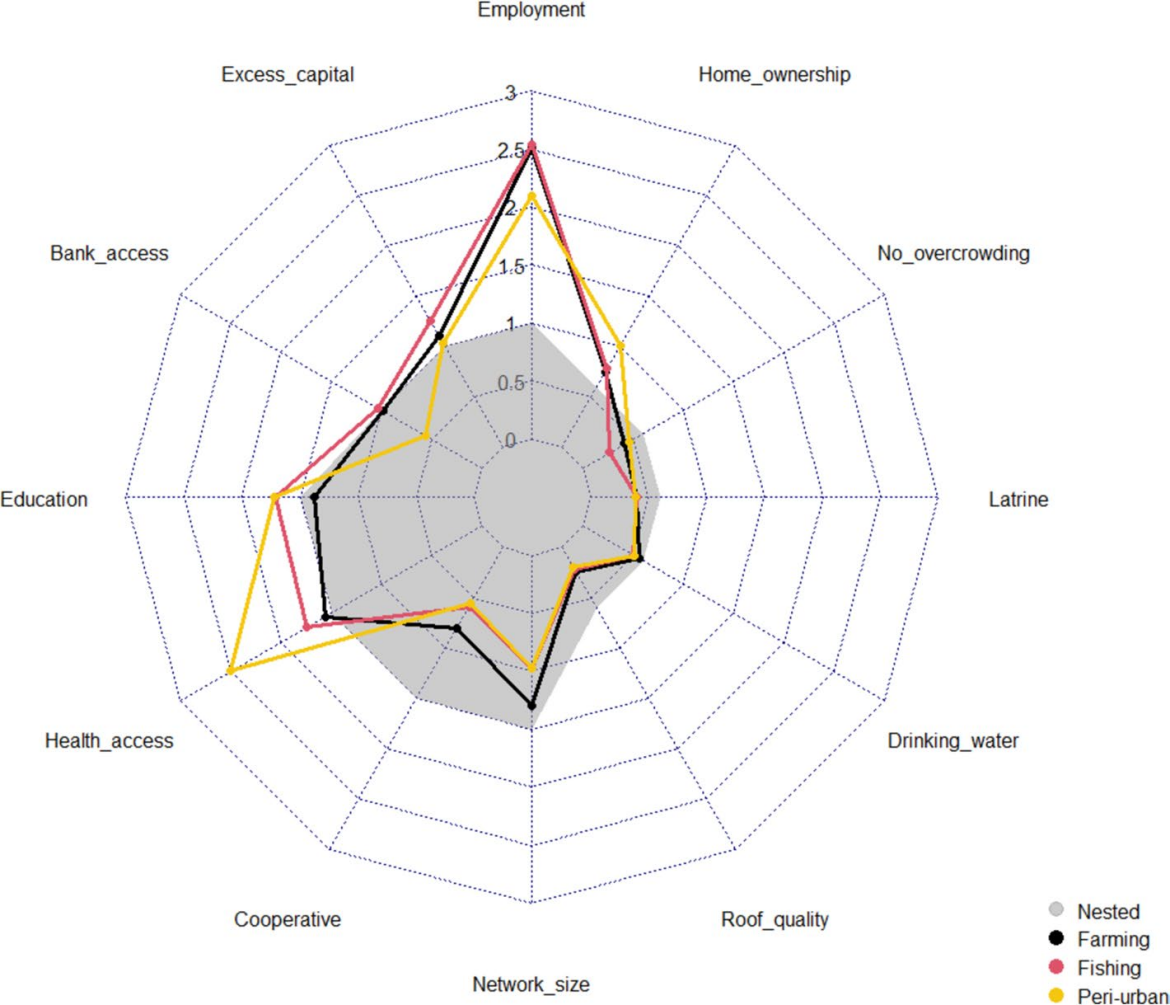
Comparison of basic needs weighted scores by livelihood group. “Nested” weights displayed as a comparative baseline

Significant differences in the scores given to “employment”, “bank access”, “healthcare access”, “cooperative membership”, and “network size” were found between livelihoods (Fig. 3, Table 7).

**Table 7:** Test for stochastic dominance between livelihood groups’ weightings, for deprivation components with a significant Kruskal–Wallis result

		Livelihood group		
Deprivation component	Livelihood group	Farming	Fishing	Kruskal–Wallis test statistic
Employment	Fishing	0.143		7.456**
	Peri-urban	2.589***	2.437***	
Bank access	Fishing	-0.334		13.307***
	Peri-urban	3.317***	3.566***	
Healthcare access	Fishing	-1.479*		15.520***
	Peri-urban	-4.165***	-2.844***	
Cooperative membership	Fishing	1.628*		14.605***
	Peri-urban	4.033***	2.586 ***	
Network size	Fishing	2.679***		6.980**
	Peri-urban	1.416*	-0.905	

For each pairwise comparison, the z-test statistic and p-value significance are presented. A positive z-test statistic corresponds to a larger mean ranked score within the column group compared to the row group

*** p < 0.01, ** *p *< 0.05, * *p * < 0.1

Farming and fishing communities weighted “employment”, “bank access”, and “cooperative membership” significantly higher than peri-urban communities, whereas peri-urban communities scored "healthcare access" significantly higher. “Family/friend networks” were also valued significantly higher amongst farming communities than fishing groups.

When comparing the proportion of households experiencing objective basic need deprivations, significant differences were found for “bank access”, “healthcare access”, “roof quality”, “drinking water”, “latrine”, and “homeownership” (Table 8). “Bank access”, “healthcare access”, and “drinking water” deprivations were significantly higher in farming/fishing communities. The proportion of households with “latrine” deprivation was significantly higher amongst visited fishing communities, whereas “roof quality” deprivation was significantly lower. “Homeownership” deprivation was also significantly higher in visited peri-urban communities.

**Table 8:** Test for stochastic dominance between livelihood groups, with a significant difference (Kruskal–Wallis) in the proportion of households experiencing objective basic need deprivations

		Livelihood group		
Deprivation component	Livelihood group	Farming	Fishing	Kruskal–Wallis test statistic
Bank access	Fishing	1.674**		13.016***
	Peri-urban	3.705***	2.262**	
Healthcare access	Fishing	5.587***		49.710***
	Peri-urban	7.588***	2.784***	
Roof quality	Fishing	5.627***		30.533***
	Peri-urban	0.669	-4.153***	
Drinking water	Fishing	5.745***		47.359***
	Peri-urban	7.184***	2.246**	
Safe latrine	Fishing	-4.904***		36.241***
	Peri-urban	1.875**	6.073***	
Home ownership	Fishing	-1.663**		19.961***
	Peri-urban	-4.658***	-3.222***	

For each pairwise comparison, the z-test statistic and p-value significance are presented. A positive z-test statistic corresponds to a larger mean ranked score (higher deprivation) within the column group compared to the row group

### Gender

**Fig.4:**
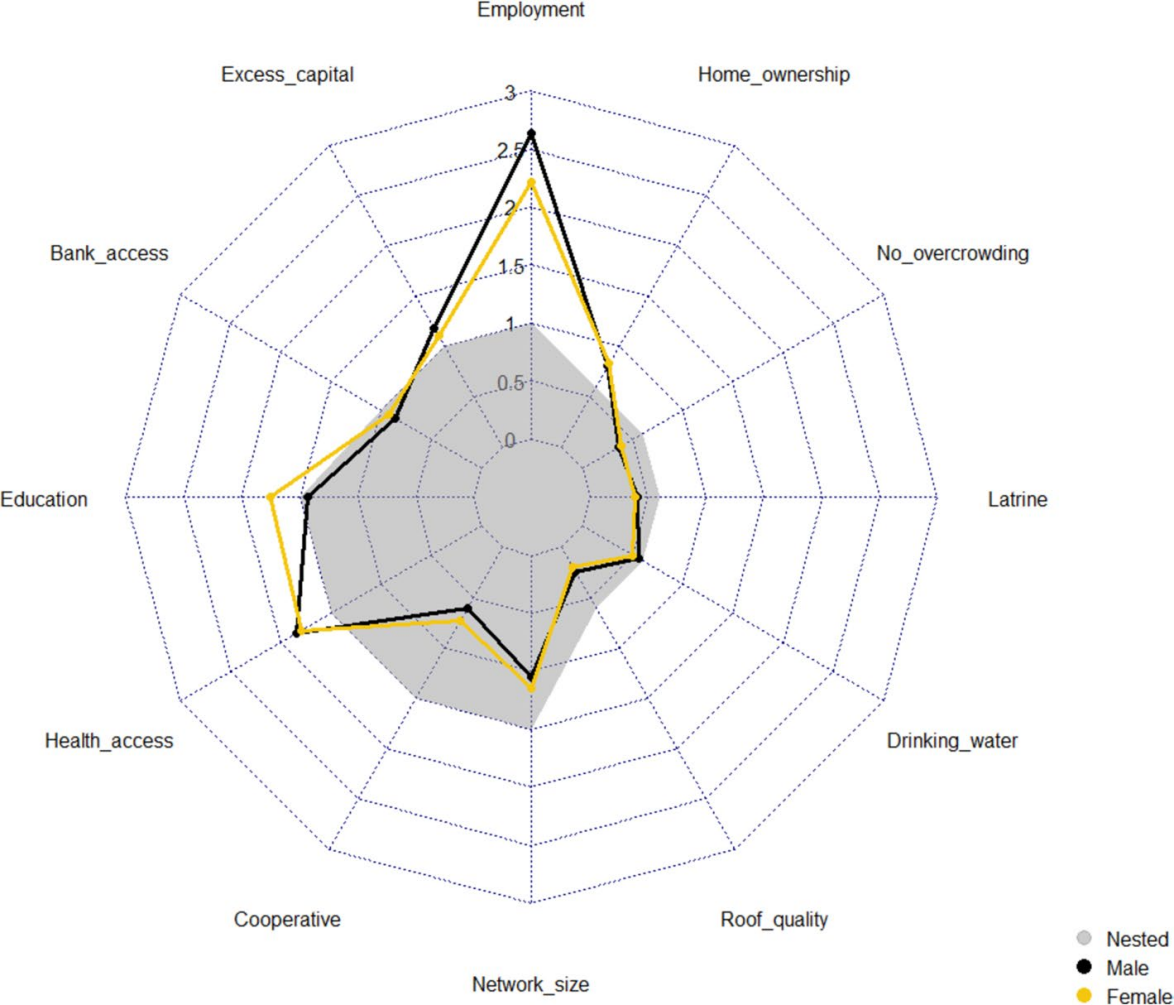
Comparison of basic needs weighted scores by gender. “Nested” weights displayed as a comparative baseline

There was relative consistency between males’ and females’ scores (Fig. 4). Only one component significantly differed, with education valued higher by female respondents (Mann–Whitney test statistic (*W*) = 1433.0***). In contrast, males applied a greater weight to “employment” (+ 0.42); however, the mean ranked difference was non-significant at the 5% level (*W* = 2337.0*).

### Decision-Making Level

**Fig.5:**
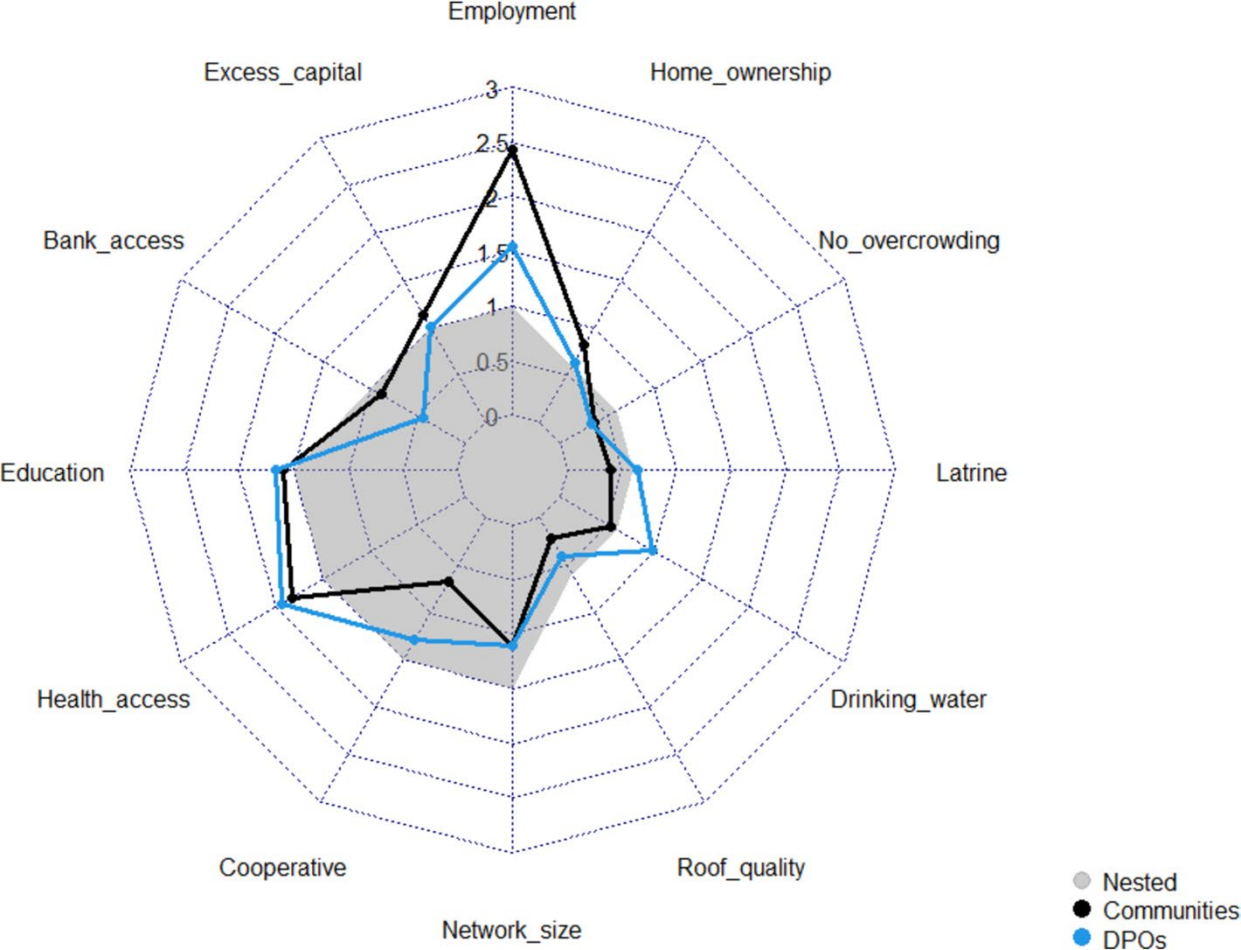
Comparison of basic needs weighted scores by decision-making level. “Nested” weights displayed as a comparative baseline

Two financial capital components, “employment” (*W* = 737.5**) and “bank access” (*W* = 727.0**), were weighted significantly higher by community members than DPOs (Fig. 5). In contrast, human capital indicators (“education” and “healthcare access”) were weighted consistently. Social capital displayed variation with “cooperative membership” weighted significantly higher by DPOs (*W* = 247.5**), while “network size” was weighted similarly. DPOs also scored 3/5 physical capital components significantly higher than communities: “roof quality” (*W* = 300.0**), “drinking water” (*W* = 133.0***), and “latrine” (*W* = 283.0**).

### Deprivation Rates

**Fig.6:**
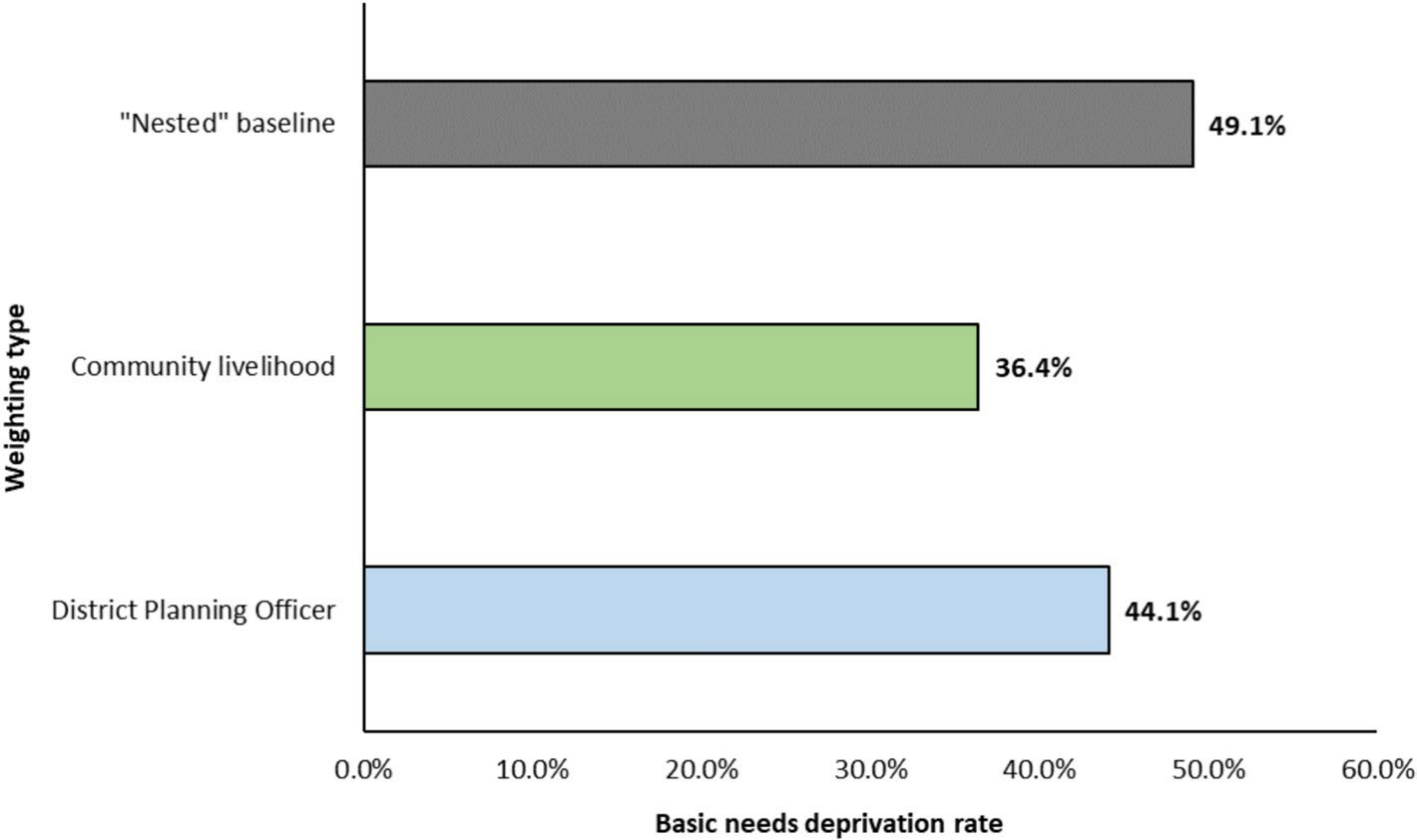
Overall basic needs deprivation rate by weighting type; “nested” baseline, community livelihood and District Planning Officer (DPO) weights

Applying different weights influences the overall deprivation rate for surveyed households in Volta Delta (Fig. 6). Community and DPO-weighted rates are 12.7 and 5.0 percentage points lower than the “nested” rate respectively. When examining livelihood groups’ rates, the difference to the “nested” rates is smaller in peri-urban communities compared to farming and fishing groups (Table 9).

**Table 9:** Basic needs deprivation rates by livelihood group classification across the 50 DECCMA survey communities. Deprivation rates and absolute/relative differences between the baseline “nested” weighting approach and community livelihood weighting

Livelihood group	“Nested” rate (%)	Community livelihood rate (%)	Absolute difference (%)	Relative difference (%)
Farming (n = 22)	52	38	− 14	− 27
Fishing (n = 16)	58	43	− 15	− 26
Peri-urban (n = 12)	32	25	− 7	− 22

A sensitivity analysis of the different livelihoods’ deprivation rates was conducted to validate the livelihood classification (Appendix I).^10^ The results demonstrate sufficient robustness, yet also highlight the importance of local-specific analysis and how other characteristics beyond livelihood may influence wellbeing priorities.

The community livelihood rate is also 7.7 percentage points lower than the DPO rate (Fig. 6), primarily driven by the greater prevalence of “cooperative membership” deprivation (Table 6) and the higher weight applied by DPOs (Fig. 5). Examining differences across livelihood groups, there is greater alignment between peri-urban community and DPO-weighted rates (Table 10).

**Table 10:** Basic needs deprivation rates by livelihood group classification across the 50 DECCMA survey communities. Deprivation rates, and absolute/relative differences, between the DPO weighting approach and community livelihood weighting

Livelihood group	DPO rate (%)	Community livelihood rate (%)	Absolute difference (%)	Relative difference (%)
Farming (n = 22)	48	38	− 10	− 21
Fishing (n = 16)	51	43	− 8	− 16
Peri-urban (n = 12)	26	25	− 1	− 4

Applying weights from different livelihoods to the 50 surveyed communities produces varying deprivation rates (Table 11). Farming and peri-urban communities show relatively small differences, with rates ranging 3% and 1% respectively. The largest difference is found in fishing communities, with peri-urban weights producing a deprivation rate 7% higher than the fishing-weighted rate. This difference is attributable to the greater number of fishing households experiencing “healthcare access” deprivation (94%)^11^ and the significantly higher weight applied by “peri-urban” communities (Fig. 5).

**Table 11:** Basic needs deprivation rates by livelihood group classification across the 50 DECCMA survey communities, applying the weightings from the three visited community livelihood types

Livelihood group	Farming-weighted rate (%)	Fishing-weighted rate (%)	Peri-urban weighted rate (%)	Range (%)
Farming (n = 22)	38	39	41	3
Fishing (n = 16)	42	43	49	7
Peri-urban (n = 12)	24	24	25	1

### Spatial Distribution

This section focuses on the spatial distribution of basic needs deprivation across Volta Delta when applying community-level livelihood weights, compared to baseline “nested” and DPO weights.

**Fig. 7:**
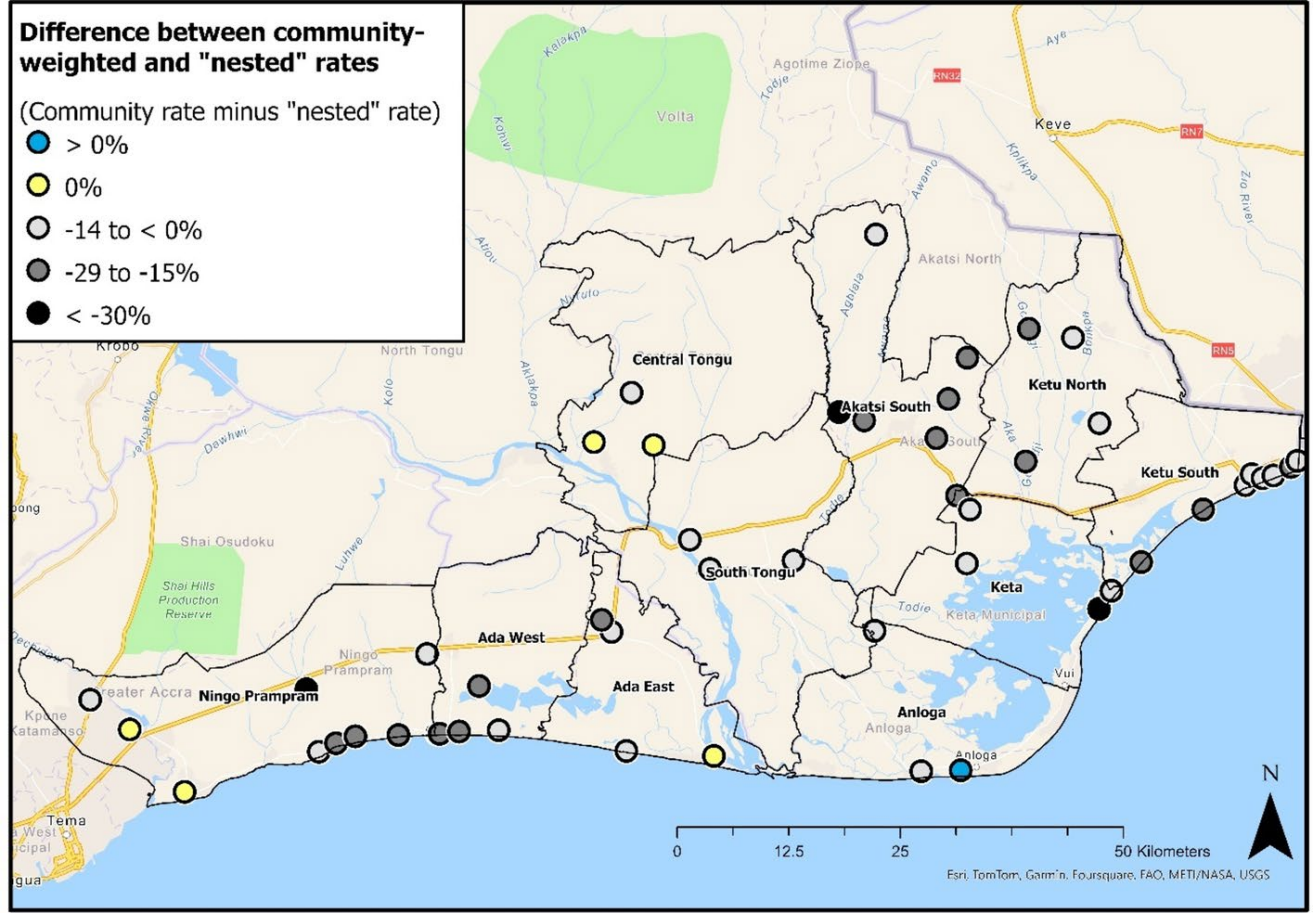
Distribution of community-level differences between “nested” baseline and community livelihood-weighted deprivation rates. Values are calculated by subtracting the baseline rate from the community rate

When applying community livelihood weights, 44/50 surveyed communities have a lower deprivation rate than when “nested” weights were used. On average these communities record a rate 14 percentage points lower. In contrast, one location has a lower rate when using “nested” weights, and five communities show no difference (Fig. 7). However, examining individual communities illustrates greater sensitivity to weighting selection. For example, three communities record substantially lower deprivation rates (> 30%) when using community weights compared to “nested” weights. These differences are driven by the greater “nested” weight applied to “cooperative membership” (Fig. 3) and the large proportion of households not accessing cooperative groups (Table 6).

A clear spatial pattern does not appear when exploring differences between baseline and community livelihood rates (Fig. 7). For example, the largest differences (> 30%) are in three separate districts: Ningo Prampram, Akatsi South and Keta. Yet, lower differences are primarily located near built-up landscapes. For example, similar community-weighted and “nested” rates are observed in west Ningo Prampram near Tema, Central Tongu, and South Tongu. This finding is supported by the lower average difference in community-level deprivation for peri-urban communities (Table 9).

**Fig. 8:**
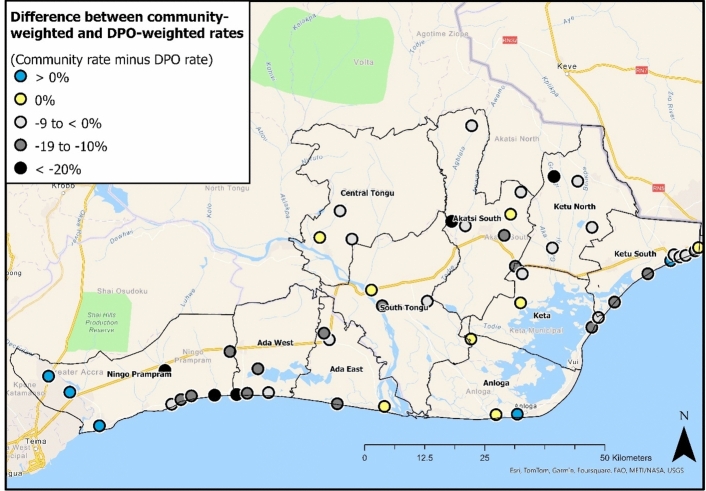
Distribution of community-level differences between DPO and community livelihood-weighted deprivation rates. Values are calculated by subtracting the DPO rate from the community rate

Next, comparing community livelihood and DPO rates, 37/50 locations have a lower deprivation rate when applying community weights. On average these communities record a rate 11 percentage points lower. In contrast, five locations have a lower DPO-weighted rate, and eight communities show no difference (Fig. 8). Again, examining individual communities illustrates greater sensitivity. For example, five communities record substantially lower deprivation rates (> 20%) when applying community livelihood weights. These differences are primarily driven by the greater DPO weight applied to “cooperative membership” and “drinking water” deprivations (Fig. 5). See Appendix J for further maps illustrating differences in community and DPO-weighted incidences^12^ of “employment”, “cooperative membership” and “drinking water” deprivations; the three indicators with the largest differences in community/DPO weighting.

Similar to community and “nested” comparisons, the communities with the largest differences between DPO and community-weighted rates do not show a distinct spatial pattern (Fig. 8). The five communities with > 20% lower community-weighted rates are located across four districts: Ningo Prampram, Ada West, Akatsi South and Ketu North. However, a cluster of communities with lower DPO-weighted rates is found close to Tema (Ningo Prampram). All three communities are “peri-urban”, with the higher community-weighted rate attributable to higher-than-average proportions of “employment”, “health access”, and “homeownership” deprivations; all weighted higher by peri-urban respondents than DPOs. In contrast, no households within these three communities experienced “drinking water” deprivation, dampening the influence of the significantly greater weight applied by DPOs (Fig. 5).

## Discussion

This section, structured using the SLF objective capital types, draws on community FGs and DPO interviews to explore potential reasons for the different weightings across livelihood, gender and decision-making subgroups. Nevertheless, it is recognised how the differences in weighting scores between the various socioecological subgroups may simply reflect differences in their values and conceptualisations of a “good life”. The influence of weighting selection on the overall deprivation rate and spatial distribution is also reviewed.

### Financial Capital

Comparing livelihoods, the greater weight applied to “employment” amongst farming and fishing communities (Table 7) aligns with the importance of employment in fulfilling intergenerational obligations and identities (Béné & Friend, 2011; Brown et al., 2021; Markussen et al., 2018), and achieving food security in primarily subsistence locations (Asare-Nuamah, 2021).

“I am proud to be a farmer because our work brings food to the community…Farming is the only source of happiness, we are content” **(5,FG,M)**

“employment is more important to us here…if I’m not working then I don’t eat” **(2,FG,M)**

The greater weight applied to all financial components within farming/fishing communities contrasts theories which suggest primary-based communities place less emphasis on financial OWB when defining their “good life” compared to money-orientated peri-urban communities (Guillen-Royo & Velazco, 2012; Osei-Tutu et al., 2020; Theodoropoulos, 1999; Veenhoven, 1993). However, this could also reflect a bidirectional effect, as rural landscapes with lower land costs may have attracted individuals with lower financial OWB (Appendix K) and more immediate requirements for employment to provide basic needs for their family and community (Kansanga et al., 2022; Moseley, 2001). In contrast, more affluent, peri-urban communities may have less short-term insecurities and therefore focus on long-term wellbeing challenges such as improved formal healthcare (Fig. 3). This hypothesis is supported by studies illustrating that greater individual wealth often results in higher health expenditure to increase life expectancy and life satisfaction (Hall & Jones, 2007).

Farming/fishing communities also weighted “bank access” significantly higher than peri-urban communities (Table 7) and DPOs (Fig. 5). This result potentially reflects the lower availability of formal banking services (Table 8) and the greater requirement for credit access to overcome seasonal income fluctuations (Afful et al., 2015). Landscape characteristics within rural communities, including poor road networks, can also limit physical accessibility to financial services. Ghanaian studies suggest banks are reluctant to lend to remote households due to difficulties in collecting repayments (Asiedu et al., 2013; Porter, 2007), with limited credit access a crucial determinant of poverty entrapment (Banerjee & Jackson, 2017). Limited accessibility to markets within rural communities may also reduce trading profits (Owusu & Lund, 2004), lowering access to the excess capital required to start saving within banks.

“we usually spend all our money on taking care of the home…we do hand to mouth with the income. The desire is not there to save money” **(4,FG,M)**

FG respondents may have used the exercise to emphasise the need for new institutions and services, potentially influenced by our positionality and the awareness of our engagement with governmental decision-makers (Dosu, 2021). This hypothesis is supported by higher proportions of “bank access” deprivation within farming/fishing communities (Table 8), suggesting that in this situation individuals placed higher weights upon components they currently wanted, but did not have access to. This possible disconnect between longer-term financial aspirations and the required skills and infrastructure needed to achieve these goals was illustrated in Nyitawuta (Site 4).

“the road…is not good, we do not have access to electrical power so it is not conducive for a bank to be established here, but we have small susu association where at the end of the year we count and distribute it” **(4,FG,M)**

In contrast, peri-urban communities potentially viewed banking services as “normality” and therefore less crucial to their immediate “good life”. This result is supported by the lower proportion of peri-urban households experiencing “bank access” deprivation (Table 8).

“when you make money, you can pay for your light bill, water bill and take care of your home…the little you have you can save in the bank” **(1,FG,F)**

Next, examining gendered differences, the male “employment” weight was higher, yet non-significant at the 5% level. This finding adds to discussions regarding changing gendered responsibilities in Ghana (Vercillo, 2020). The higher “employment” score supports traditional male “breadwinner” identities (Tolhurst et al., 2008). Greater resource access and involvement in community decision-making among male community members (Lambrecht, 2016; Ramón-Hidalgo & Harris, 2018) are crucial in ensuring that households’ needs, such as food security and healthcare, are achieved. Fulfilling these “masculine” obligations through employment is important for male SWB, with Central Region studies highlighting the embarrassment caused if female members contribute more towards household finances (Addai et al., 2015; Carr, 2008).

“the inability to take care of your wife and children causes heartache to the man of the house” **(5,M,FG)**

However, the non-significant difference alludes to changing gendered dynamics, with Northern Region studies illustrating male labour to increasingly focus on subsistence, while female labour targets income generation (Vercillo, 2020). Female employment is suggested to be progressively important in dampening seasonal financial fluctuations, with “male” seasonal agriculture/fishing accompanied by diverse, year-round “female” activities (Tolhurst et al., 2008). The consistent scoring between genders also provides further evidence that the weighting exercise methodology effectively limited the influence of key community members upon others’ weighting decisions.

“we are able to do petty jobs but men are not able to…They want to get money at once but…we search for little ones and when it is needed we are able to fall on them…to make money” **(4,CI,F)**

Furthermore, DPOs recorded a significantly lower “employment” score than communities (Fig. 5). This potential underestimation is exemplified in Anyamam (Site 2), where despite traditional small-scale salt mining livelihoods being prohibited following the sale of Songor Lagoon to private investors (Harvey & Langdon, 2010; Roland et al., 2019; Yeboah et al., 2013), the district government created no alternative livelihoods. This resulted in violent conflict between resistant communities and privately-funded police (Agbove, 2022), and has reduced communities’ incomes and perceived security (Vladisavljević & Mentus, 2019).

“[restricted access to Songor Lagoon]… has affected their livelihood, it was a quick source of money, they depended highly on it…now that way of making money has been stopped…the disadvantage was that we did not put any alternative livelihood measures in place” **(2,DPO)**

The difference between the decision-making levels is particularly pronounced within primary sector communities, with fishing and farming communities weighting “employment” significantly higher. Therefore, using DPOs’ weights within development policy could lessen the focus on fishing/farming communities, with the highest levels of unemployment located north of Keta Lagoon and in coastal Ningo Prampram (Appendix J.1).

Nevertheless, despite the disparity in scores, DPOs did emphasise the importance of employment for a “good life”. The lower score could be attributed to the different responsibilities of communities and DPOs. For example, as DPOs coordinate and monitor development plans across various government institutions, they could interpret “basic needs deprivation” from a broader perspective to a community member who may have a narrower set of immediate challenges. This theory is supported by the smaller range of scores given to overarching capitals by DPOs, compared to communities (Appendix L.1), and the stronger alignment between DPO weights and baseline “nested” weights, which assume equal importance across the objective capital types (Appendix L.2).

The greater emphasis on “employment” by communities, compared to DPOs (Fig. 5), may stem from their direct awareness of how occupations facilitate access to wide-ranging wellbeing capitals and act as conduits for SWB by fulfilling cultural identities (Brown et al., 2021; Markussen et al., 2018). Many respondents noted that before they could consider accessing other basic needs, employment and a source of income were required. Therefore, community members may view employment as encompassing other needs, whereas DPOs may have interpreted different components independently due to them being addressed by separate institutions.

### Human Capital

Peri-urban communities scored “healthcare access” significantly higher than farming/fishing communities (Fig. 3), primarily driven by Sogakope (Site 3) (Appendix H). This finding initially appears counter-intuitive, with both visited peri-urban communities having lower “healthcare access” deprivation (Table 8).

“we are lucky, we have clinics around here…and we are close to Tema general hospital, so when someone is…referred…it’s easy” **(1,FG,M)**

However, this emphasises how wellbeing perceptions form within the “relational context” (White, 2016), with peri-urban communities potentially exposed to particular health challenges at the time. For example, all female FG respondents in Sogakope (Site 3) initially claimed “good health” was the only characteristic of a “good life”. The non-random selection of participants may have also contributed towards specific issues being prioritised within certain FGs. Additionally, living close to health services could raise expectations, yet if the service is low-quality or if there are non-physical barriers such as high fees (Imoro, 2015), frustrations could increase the perceived importance during the weighting exercise. This finding potentially juxtaposes studies showing that urban dwellers in Ghana are less willing to pay higher taxes for healthcare than rural dwellers with less access to high-quality facilities (Adisah-Atta, 2017).

“health insurance, you cannot use it when you do not have money”…”I have to go for check-up, but I do not have money” **(3,FG,F)**

In contrast, the lowest community-level “healthcare access” scores (Appendix H) were given by; (i) remote, agricultural Nyitawuta (Site 4), where the soon-to-open NGO clinic provided a relative improvement and future optimism regarding community’s health, potentially lowering the immediate emphasis on “healthcare access” during the weighting exercise, and (ii) Anyamam (Site 2), where limited health care, beyond “*two health centres, one clinic and five Community Health Planning Services (CHPS) for whole district [Ada West]”* **(2,DPO)** could have created an acceptance, where households become accustomed to limited services to the extent it limits their ability to “recognise…weaknesses in the system” (Amoah et al., 2021;12).

Next, examining gendered differences, female respondents scored “education” significantly higher (Fig. 4). This result supports the gendered “caregiver” role within traditional Ghanaian communities (Quaye et al., 2016). For example, female household heads spend a greater proportion of remittances on children’s education than males (Pickbourn, 2016; Teye et al., 2022).

“women sympathise with the children more than the men…because they are mostly with us” **(5,CI,F)**

### Social Capital

Social capital components, “cooperative membership” and “family/friend networks”, were scored significantly higher amongst farming communities (Table 7). This result supports theories that reciprocal relationships and place attachment are more important to a “good life” in agricultural communities (Leviston et al., 2018; Markussen et al., 2018; Schutte et al., 2022).

“a good life is someone who respects others…the person is of help to others …when the person has good health…and has a good attitude towards people” **(4,CI,M)**

The importance of social capital may be amplified within farming communities due to the potential resiliency it can provide during climatic risks, such as drought (Garrity et al., 2010; Heger et al., 2018), which can negatively affect households’ financial, subjective and psychological wellbeing (Jordan, 2015). These ideas are exemplified by individuals’ responses to the question, “do community relationships strengthen or weaken during climatic shocks?”.

“it strengthens our unity very much, we are each other’s keeper so we ensure that affected persons are taken care of” **(8,FG,M)**

“we provide support for each other in such situations…people bring up ideas [to]…help the affected person…we are very sympathetic when our neighbours are in distress” **(4,FG,M)**

However, social capital was also shown to fluctuate in response to climate risk (Craig et al., 2023), challenging the romanticised assumption of persistent togetherness and happiness within vulnerable, rural locations (Kay & Jost, 2003; Markussen et al., 2018).

“people who were not affected will hesitate in supporting the affected people because they do not know when they may also be affected” **(5,FG,M)**

In contrast, lower scores for social capital components amongst peri-urban communities could reflect greater individualism, money-orientated cultures, and higher inequality (Appendix M). Inequality, potentially driven by varying levels of adaptive capacity and access to less-vulnerable employment, can generate perceptions of otherness and inadequacy, resulting in lower SWB (Kangmennaang et al., 2019; Kingdon & Knight, 2007). Therefore, reducing the interest in social capital when defining a “good life” during the weighting exercise.

“we are one people but things are getting difficult, now we don’t have love among us anymore, we will gather, talk and laugh, but when someone is going far then others are envious” **(1,CI,M)**

Differences also exist between communities and DPOs, with “cooperative membership” scored significantly higher by DPOs (Fig. 5). Due to high levels of “cooperative membership” deprivation (Table 6), there are differences between community and DPO-weighted incidences across all surveyed communities in Volta Delta. However, the disparity is particularly pronounced within coastal locations (Appendix J.2), with peri-urban and fishing communities weighting “cooperative membership” deprivation significantly lower than farming communities (Table 7). The large disparity in scoring between DPOs and communities in these areas could influence measured wellbeing outcomes and policy interest in select communities. The difference could be attributed to DPOs being unaware of the challenges faced within community cooperatives, such as corruption and distrust (Dary & Grashuis, 2021).

“cooperative is a society on its own, not like a family, something can happen to you now and the cooperative won’t come to your aid” **(7,FG,M)**

Additionally, several DPOs mentioned the limited funding available to districts; therefore, cooperatives may have been viewed as low-cost strategies to address social issues without the need for large interventions. Previous studies allude to how local governments and international organisations can be overly dependent upon cooperatives to address social issues and stimulate growth, despite community groups functioning within wider structural systems in which they have little influence (Afranaa Kwapong & Hanisch, 2013; Simmons & Birchall, 2008). The expectation that communities should address their own social issues, such as sanitation, was explicitly referenced by one DPO.

“sanitation is a personal responsibility, but they are not getting it…they feel the government should be responsible” **(2,DPO)**

One DPO also noted how cooperatives act as channels for governmental support and can facilitate access to public funding; *“if you are in a group then government can give you equipment for planting, harvesting…So individuals suffer, but cooperative groups do not”* **(5,DPO)**.

The ability for cooperatives to access public funding, alongside potential differences in the expectations and perceived efficiency of community groups, could explain the disparity in weighting between communities and DPOs.

### Physical capital

Significant differences in physical capital scores were recorded between communities and DPOs, yet no component significantly differed across livelihood or gender subgroups. Therefore, this subsection also draws upon key community-level differences to further explore potential relationships between physical capital and wellbeing priorities across different landscapes.

Firstly, “drinking water” was weighted lowest in rural Nyitawuta (Site 4) (Appendix H). This result potentially reflects how the recent relative improvement (Ravallion et al., 2013) from the newly-constructed dam reduced the emphasis placed upon “drinking water” within the community’s conceptualisation of a “good life”.

“we walked 10–11 km before we get water to drink, even that we share with cattle…A dam was created [2020] [which]…now serves as a source of drinking water for the community…*What distance?* 4 km…we got the dam so that infection has reduced” **(4,FG,M)**

However, despite the visible joy amongst community members when water was delivered, the water was low quality. Multiple respondents discussed their challenges in accessing safe water, supported by the significantly higher proportion of farming households experiencing “drinking water” deprivation (Table 8). This finding suggests that individuals’ weighting priorities may not always reflect what they do not currently have access to, but rather how communities’ “desires…[can be] constrained by what seems possible” (Laderchi et al., 2003;253). Furthermore, despite persistent issues, improvements compared to a low comparative reference point and the capacity to freely access water, unlike in urban areas, could have reduced the current value applied to “drinking water”.

“even though sachet water is good, over here all you have to do is get 1–2 cups of water to drink, you do not have to pay for it and we are happy” **(4,FG,F)**

In contrast, Awlikope (Site 5) weighted “drinking water” highest (Appendix H). The assemblyman noted a divide between local leaders and government officials, stemming from government-supplied piped water not functioning for years. This perceived injustice, the increased dependence upon fresher yet uncertain rainwater due to borehole salinization, and the acknowledgement of DPOs being involved in the research project potentially resulted in “drinking water” being prioritised during the weighting exercise.

“[water is a] long-term issue, even the pipe the government provided, the water available is very few and far between…the politicians, they always want to line their pocket to the detriment of society” **(5,CI,M)**

However, despite this potential disconnect between communities and government, DPOs weighted “drinking water” significantly higher than communities (Fig. 5). The significant difference between decision-making levels is particularly pronounced in Akatsi South and Ketu North/South, where higher levels of “drinking water” deprivation increase the sensitivity of households’ deprivation classification to weighting selection (Appendix J.3).

One potential reason for the difference could be how many communities recently received relative improvements in their drinking water; for example, in Kedzi (Site 7) where a piped supply was installed in 2020 to reduce pressure upon increasingly saline boreholes. Therefore, lower scores may have been provided if previous relative comparison points had been improved upon (Ravallion, 2014). Furthermore, many communities had access to some form of drinking water, whether piped, borehole, sachet or open-source. Therefore, the weighting scores may have reflected what the communities desired in that moment, yet did not currently have access to, rather than what was needed for a “good life” over time. As mentioned, the scoring may have been influenced by our positionality and the perception that we were working with government agencies to provide immediate support (Dosu, 2021; Frey & Gallus, 2013).

Additionally, previous studies suggest Ghanaian communities possess a greater acceptance of challenges, such as accessing water, due to “the social reality of little environmental control and slow socioeconomic and infrastructural development” (Dzokoto, 2012;318). Temporary solutions to ongoing issues are often readily deployed, such as driving around potholes or collecting alternative water sources (Box 1). This perspective potentially resulted in certain deprivations being accepted as the “norm”, and the prioritisation of basic needs that communities felt they did not have alternative channels of access.

**Box 1:** A hypothetical conversation created by Dzokoto (2012;319) to symbolise the acceptance of social and environmental challenges in Ghana

**New to Ghana**: I turned on the tap, and there was no water!	
**Ghanaian**: Yes, the water isn’t running.	
**New to Ghana**: What do you mean the water isn’t running?	
**Ghanaian**: Like I said, the water isn’t running.	
**New to Ghana**: Well, why isn’t the water running? Is there a water main break?	
**Ghanaian**: No.	
**New to Ghana**: Well, is there a drought or something?	
**Ghanaian**: No.	
**New to Ghana**: So then why isn’t the water running?	
**Ghanaian**: My friend, this is Ghana. Sometimes, the water runs, sometimes, it doesn’t. That is how it is…Here, take this bucket. There is water in the tank around the corner.	

The cultural resiliency could also support communities' significantly lower score for “roof quality” compared to DPOs (Fig. 5). For example, respondents in Nyitawuta (Site 4) reported that free access to timber and other natural materials provided alternative solutions even when unable to afford metal sheeting.

“I am not able to change my roofing sheets because my business did not flourish …[but] this is our hometown and we have natural resources like timber, which we consider as our timber. We cannot sell, we only use it for roofing”** (4,FG,M)**

Next, despite “latrine” being weighted similarly across livelihoods, illustrating the universal importance of sanitation to communities’ development and wellbeing (Duku et al., 2022; Simiyu et al., 2022), visited fishing communities possessed greater levels of deprivation (Table 8). Furthermore, differences between community-level weights were recorded, with coastal communities Anyamam (Site 2) and Anloga (Site 8) providing two of the three lowest scores (Appendix H), despite being located in flood-prone areas where poor sanitation can exacerbate health issues (Stanke et al., 2013). This result could relate to the cultural acceptance and perceived spiritual benefits of open defecation in some coastal villages (Osumanu et al., 2019). The difference in traditional beliefs and the understanding of the health risks attributed to open defecation (Stanke et al., 2013) between communities and local government could also clarify why DPOs weighted “latrine” significantly higher than communities (Fig. 5).

“along the coast they have that belief that when you are going to the toilet the fresh air…I think it is cultural, I do not understand” **(2,DPO)**

Furthermore, the significantly higher weight applied to 3/5 physical capital components by DPOs, compared to communities, potentially illustrates their different perspective when conceptualising objective basic needs deprivation. DPOs emphasise infrastructure developments more than communities, who prioritise employment as a conduit for wider OWB. This highlights the requirement for greater capacity within local government to engage with local communities to mitigate conflicts and address local concerns.

These examples illustrate the importance of collecting weightings from different socioecological groups, as aggregating weights across space may not capture the heterogeneity of required interventions and desired outcomes. The next section explores the sensitivity of the overall basic needs deprivation rate and spatial distribution to weighting selection.

### Deprivation Rates

Different weighting approaches produce varying overall deprivation rates (Fig. 6), highlighting the sensitivity to weighting selection. Both community and DPO weights produce overall deprivation rates lower than the baseline method, suggesting “nested” weighting overestimates basic needs deprivation within the context of Volta Delta. This sensitivity also illustrates how weighting selection could impact the effectiveness of policies with limited resources. For example, if the ten most deprived communities using “nested” or DPO weighting were selected for a wellbeing initiative, two communities within the ten most deprived when using community weighting would be excluded (Appendix N). These two farming communities are overrepresented amongst both “employment” and “excess capital” deprivations; therefore, “nested” or DPO weighting could result in financially vulnerable, and potentially food insecure, communities being omitted from policy intervention. This scenario emphasises the need to examine different weighting approaches within wellbeing research to ensure the most vulnerable communities are targeted (Booysen, 2002).

Community-weighted livelihood rates are substantially lower than “nested” deprivation rates across most communities. However, the spatial pattern is not well-defined, with nearby communities experiencing varying effects depending on weighting selection (Fig. 7). These small-scale differences emphasise wellbeing's “relational context” (White, 2016). However, a lower average difference in overall deprivation amongst peri-urban communities, compared to farming and fishing groups (Table 9), suggests in certain contexts, assuming equal “nested” weighting may not always substantially overestimate basic needs deprivation.

Similarly, most communities record higher deprivation rates when applying DPO rather than community livelihood weights (Fig. 8). This result is primarily driven by the greater scores applied to “cooperative membership” and “drinking water” deprivations by DPOs. These differences are pronounced in different locations, with more frequent “cooperative membership” deprivation and lower community weights within coastal communities (Appendix J.2), whereas higher levels of “drinking water” deprivation, weighted higher by DPOs, are concentrated within inland farming communities in Akatsi South and Ketu North (Appendix J.3).

Viewing these differences across livelihoods, there is a greater alignment between DPO and community-weighted rates in peri-urban communities (Table 10). In contrast, larger differences in “overall” deprivation rates are found in farming and fishing communities, which also possess comparatively higher levels of objective basic needs deprivation. Therefore, DPOs’ perceptions of communities’ needs, particularly regarding financial capital, could be most disconnected from communities with the greatest challenges. However, lower sensitivity to weighting selection does not mean that similar priorities, and therefore policy targets, exist between DPOs and peri-urban communities. For example, the larger weight applied to “employment” by peri-urban communities is offset to an extent by the higher DPO weight given to “cooperative membership” (Appendix L.2). Therefore, as well as illustrating the sensitivity of wellbeing outcomes to weighting selection, this study highlights the capacity for weighting exercises and decomposable measures to facilitate more targeted policies that better align with communities’ priorities.

Next, when applying different livelihood groups’ weights to the entire survey sample, the range of deprivation rates is relatively narrow (Table 11). The exception being the greater deprivation rate amongst “fishing” communities when applying “peri-urban” weights, driven by the higher weight and prevalence of “healthcare access” deprivation (Tables 6and7). This finding suggests communities with greater access to certain basic needs may value those elements more than those with less access. However, since “healthcare access” is a proxy measure based on “distance to hospital” (Appendix B), peri-urban respondents’ higher weighting might reflect frustrations with non-physical barriers to access, such as unaffordable medical fees.

Nevertheless, despite the relatively small differences in deprivation rates across livelihoods (Table 11), the weighting comparison (Fig. 3) illustrates that the subgroups’ priorities *do* differ. Therefore, similar overall rates might result from different weights offsetting one another. These findings underscore the importance of collecting weights from diverse locations and socioecological subgroups to avoid assuming that one group’s values are universally applicable and to discourage blanket development initiatives.

Overall, community-preference weights and consequent deprivation classifications are not homogenous across socioecological subgroups. Therefore, wellbeing research should aim to collect various groups’ wellbeing priorities. Future research should revisit all communities to ensure up-to-date livelihood group classifications.

## Conclusion

This paper illustrates how subjective perceptions can be incorporated within OWB measures, and how wellbeing priorities vary between socioecological groups in Volta Delta. Significant differences were observed in how livelihood groups conceptualised a “good life”. For example, farming households placed higher value on “employment” and social capital, reflecting the role of agricultural labour in fulfilling social obligations and the importance of collective wellbeing within rural landscapes. Conversely, peri-urban communities weighted “healthcare access” higher, potentially highlighting the frustrations with non-physical barriers, as physical accessibility was comparatively high.

Differences between decision-making levels, most notably with “employment” and “cooperative membership”, indicated a disconnect between communities and local government. Therefore, the weighting exercise could be an effective policy tool, highlighting to local decision-makers the discrepancies between their perceptions and communities’ lived priorities. This exercise could be particularly powerful within Ghana, where development strategies have traditionally been top-down economic initiatives, with limited scope for including local knowledges (Domfeh & Bawole, 2009).

Applying various community and expert-preference weights highlighted the sensitivity of wellbeing classification to weighting selection. Deprivation rates for all three livelihood groups were lower with community weights compared to “nested” weights, illustrating the limitation of applying externally derived weights to multidimensional measures. However, applying subjective weights to an “objective-list” of basic needs may still misrepresent communities’ main challenges. Future research should first capture communities’ prioritised basic needs *before* collecting weights. Moreover, this study shows “where and whom you collect weightings from *matters*”; therefore, future research should also aim to collect and apply weightings at the household level to accurately represent individuals’ concerns, and avoid assuming a single livelihood homogenously represents each community.

This study also highlighted how certain individuals might allocate weights based on their immediate needs, for which they had no alternatives, rather than what is needed generally for a “good life”. Future work should address these internal biases by refining the methodology. For instance, the weighting exercise could be reframed to ask respondents what constitutes a “good life” in a hypothetical village rather than their own. Accompanying this with other PRA methods, such as asking respondents to sketch their “ideal” community (Schreckenberg et al., 2016), could further encourage participants to think more generally.

## Supplementary Information

Below is the link to the electronic supplementary material.

Supplementary file1 (DOCX 4560 KB)

## Appendix

Please find appendices within the online Supplementary Information.

## Footnotes

1 Enumeration areas were selected proportional-to-size, and are designed to have similar household counts (n = 150) (Cordes et al., 2021). Therefore, it was assumed households had approximately equal selection probabilities (Groves et al., 2011).

2 Only one semi-structured interview was carried out in Site 1 (Afienya) due to time restraints and two potential participants dropping out early in the interview process.

3 Due to availability, two males were interviewed in Site 7 (Kedzi).

4 If the DPO interview was undertaken on the telephone (2/8 interviews) the respondent was asked to distribute ten “hypothetical” sticks. The methodology was similar, the only difference being that the DPOs contacted by telephone did not have access to the supporting pictograms.

5 Verbatim quotes are classified by the site number (Table 2), and whether the quote was said during a community focus group/community interview/district planner interview (FG/CI/DPO). Community focus groups/interviews are also defined by gender (M/F). For example, a female focus group in Afienya would be denoted by (1,FG,F).

6 The strongest correlation (0.28) is between “healthcare access” and “latrine”. However, only five (out of 66) combinations of the binary deprivation indicators show a “strong” correlation (Appendix F).

7 Defined in the SLF as “the skills, knowledge, ability to labour and good health and physical capability important for the successful pursuit of different livelihood strategies” (Scoones, 1998, p.8).

8 Mann–Whitney tests compare 2 groups, Kruskal–Wallis tests compare 3 + groups.

9 Landcover information was created from LANDSAT-7 30m resolution images, using FAO Land Cover Classifications (Jayson-Quashigah, 2016).

10 The livelihood group deprivation rates were recalculated, and compared to the original rates, after omitting the three surveyed communities with the largest average distance from the visited communities of the same livelihood type.

11 Compared to 68% amongst peri-urban communities.

12 To compare the influence of different weightings upon specific indicators across communities, the deprivation “weighted incidence” refers to the *community livelihood weight multiplied by the number of households in the community experiencing the deprivation*.

## Declarations

### Data deposition information

The DECCMA household survey dataset is openly available at Mendeley Data (https://data.mendeley.com/datasets/223z53kwnm/1). The land cover classification data is also openly available at Delta-Portal (https://www.delta-portal.net/geonetwork/srv/eng/catalog.search;jsessionid=17A16671A3C85ABEA1519F9C670B57E4#/metadata/86b279b8-3cc9-4584-a8bd-9d4300a028df).
