# Supplementary material for: “Where and Whom You Collect Weightings from Matters…” Capturing Wellbeing Priorities Within a Vulnerable Context: A Case Study of Volta Delta, Ghana
Source: Soc Indic Res. 2025 Mar 11;177(2):863–908. doi: 10.1007/s11205-025-03524-x (PMC11993479; doi:10.1007/s11205-025-03524-x)
Supplement: Supplementary file 1 — Supplementary file1 (DOCX 4560 KB) [file 11205_2025_3524_MOESM1_ESM.docx]

**“Where and whom you collect weightings from *matters*…” Capturing wellbeing priorities within a vulnerable context: a case study of Volta Delta, Ghana**

**Appendix A** Selection of development indicators across the 10 regions of Ghana. Data from the 2010 Population and Housing Census (GSS, 2013), sourced from (Awanyo & Attua, 2018).

|  | **Region** | | | | | | | | | |
| --- | --- | --- | --- | --- | --- | --- | --- | --- | --- | --- |
| **Indicator** | **Greater Accra** | **Ashanti** | **Eastern** | **Central** | **West** | **Volta** | **Brong-Ahafo** | **North** | **Upper East** | **Upper West** |
| % regional population 12+ years with mobile phone 2010 | 73.5 | 56.1 | 44.5 | 44.9 | 46.4 | 37.3 | 40.2 | 22.3 | 24.2 | 21.7 |
| Population 12+ years using the internet (% national total) 2010 | 42.3 | 22.5 | 6.8 | 7.9 | 7.9 | 3.9 | 4.0 | 2.4 | 1.2 | 1.1 |
| Dwelling units with access to pipe-borne water (% regional total) 2010 | 64.3 | 51 | 34 | 53.4 | 46.3 | 45.3 | 37 | 28 | 18 | 21.1 |
| % urban, 2010 | 90.5 | 60.6 | 43.4 | 47.1 | 42.4 | 33.7 | 44.5 | 30.3 | 21.0 | 16.3 |
| Net migration rate (per 1000) 2010 | 324.8 | 0.6 | -127.2 | -110.1 | 120.2 | -258.6 | 51.5 | -135.5 | -257.2 | -302.5 |
| % households with electricity 2010 | 87.1 | 73.6 | 58.5 | 66.1 | 65.0 | 49.6 | 53.8 | 36.1 | 24.1 | 30.9 |
| Population per doctor (public/  private) 2012 | 3,540 | 9,715 | 19,748 | 22,505 | 27,775 | 24,728 | 15,705 | 19,163 | 39,697 | 40,502 |
| % regional trunk roads classified as good 2007 | 83.0 | 70.8 | 53.7 | 69.6 | 62.5 | 47.5 | 54.0 | 59.0 | 46.9 | 23.0 |
| Illiteracy rates (% regional population 11+ years) 2010 | 10.7 | 17.4 | 19 | 21.8 | 23.6 | 26.5 | 30.2 | 62.8 | 52.5 | 53.8 |
| % regional population 6+ years with education beyond basic education levels 2010 | 32.3 | 19.7 | 14.1 | 16.0 | 16.5 | 13.5 | 13.4 | 8.5 | 9.1 | 9.9 |

**Appendix B** Descriptions of the individual basic needs indicators, and the deprivation thresholds.

Financial (employment, excess capital & bank access)

Employment deprivation was categorised by whether a household had an unemployed member. Unemployment is defined as those who are not employed, yet have actively sought after paid employment. The DECCMA dataset did not provide information on whether individuals were actively seeking work, therefore certain assumptions had to be made. In Ghana, the working population is defined as those aged 15 (Baah‐Boateng, 2013) to a retirement age of 60 (Tawiah, 2011). However, occupation data was only collected for those aged 18+. Also, data showed that a large proportion of over 60s still work. Therefore to avoid overestimating unemployment by assuming all those aged 60+ were not working, unemployment was looked at across all ages 18+. An individual was defined as unemployed if they reported themselves as unemployed and were aged 18+ years old, not a student, retired or an unpaid carer. A household was seen to be deprived if there was at least one unemployed individual. If no one in the household was of working age or economically active (retired, student or carer) then the household was deemed to be deprived also as there would be no incoming income channel. 48 households had all working age members unemployed, however this potential deprivation cut-off was deemed too constrictive and would ignore the financial effort and burden that having an unemployed household member can have on the household as a whole (Kassa, 2012).

The second financial capital indicator captures households’ proportion of expenditure on food (excess capital indicator); reflecting sensitivity to food price changes. This study uses a cut-off of 60% total expenditure to define households as financially and nutritionally insecure; as defined by the World Food Programme and other food security and welfare studies (Junaedi, 2021; Lele et al., 2016; Rose et al., 2013). Other studies suggested a threshold of 65-75% for high levels of insecurity and 50-65% for medium levels (Hjelm et al., 2016; World Food Programme, 2017). Nevertheless, 60% is selected in this study as the mean % expenditure on food in the sample is approximately 60%; therefore a 60% threshold was deemed appropriate to identify those who spend a higher-than-average amount relative to the rest of Volta Delta, and are also classified as “insecure” by international guidelines.

Thirdly, Peachey & Roe (2004) state that access to financial services should be viewed as much of a basic need in LMICs as access to water, education and health services. Availability and access to financial institutions is also interpreted as a freedom, essential for poor communities to protect themselves and achieve socio-economic development (Bayulgen, 2013; Pogge, 2005). Without financial services, it is “harder to build up reserves, let alone use credit, insurance and other complex formal financial tools," which can aid access to wider basic needs and services (World Bank, 2012). A household is defined as deprived if they have no access to banking services or loans. Therefore, if they stated that they had access to any of the following, they were not deprived; micro finance organisations (including rural banks), informal money lenders, kinship loans or large formal banks.

Human (education & health)

Basic education in Ghana is a combination of primary school and lower secondary school, with the latter starting at age 12 and concluding after 3 years (UNESCO, 2012). Upper secondary school and higher education are deemed “additional education”; upper secondary starts at age 15 for 4 years, with higher education commonly starting at age 19. Each individual within each household, aged 15 or over, was deemed deprived of education if they had not completed lower secondary school (basic education). A household is deemed deprived of education if every household member aged 15 and over has not completed a basic education.

The second human capital indicator is “healthcare access”. Due to the common absence of objective health data within social surveys, this proxy measure was calculated as a contextual variable for each of the 50 enumeration areas, with the distance to a hospital taken from the central point of each enumeration area. This measurement is a frequently used methodology, where distance is assumed to capture information on access to healthcare and health outcomes (Aboaba et al., 2023; Dotse‑Gborgbortsi et al., 2020, 2022; Simeos & Almeida, 2014). Locations of hospitals were obtained directly from the Ghana Health Service (GHS). Using Google API, distances, based on car travel across the road network, from the community centroids to the nearest hospital were produced. The World Health Organisation (WHO) recommends that households should be within 5km of a health facility (Ashiagbor et al., 2020); therefore, a 5km cut-off was used to define households as having poor access to health facilities. Hospitals, rather than all primary/secondary health facilities, were selected due to greater data completeness, and an acknowledgment that hospitals provide a wider range of crucial health services, and are commonly provided with higher quality (Dotse-Gborgbortsi et al., 2023). Hospitals were also selected, over other health clinics and facilities, due to findings from qualitative fieldwork, where both communities and DPOs primarily discussed hospitals when questioned on health service access. Despite WHO defining the 5km threshold in relation to all healthcare facilities, many existing studies in Ghana and SSA use the same recognised threshold when exploring access to hospitals (Dickens et al., 2014; Hanson et al., 2015; Nsiah et al., 2024). To ensure consistency with these studies, and to capture the communities most deprived regarding access to quality care, the 5km threshold was maintained in this analysis. However, it is recognised that some communities will potentially have better access to primary healthcare facilities which provide basic services.

Social (cooperative membership & network size)

The first social metric is based on whether households had joined a community cooperative group in the last 5 years. If the respondent responded “no” then they are defined as deprived in objective social capital (Francesconi & Wouterse, 2011). The limitation of some households existing within a cooperative network prior to the 5-year timeframe is acknowledged. However, restricting the question to the last 5 years increases the probability that the cooperative networks are still being accessed.

The second social metric captures households’ network size. This proxy sums the number of family members from outside the household (i.e., number of uncles/aunts, cousins, nieces/nephews, brothers/sisters in-laws), and the number of friends with migration experience. Many studies reflect the role of network size in positively influencing innovation, exposure to diverse information/knowledge, reducing transaction costs, increasing new opportunities, mobilizing greater communication and broadening worldviews (Abbasi et al., 2014; Rojas et al., 2011; Zheng, 2010). It is acknowledged that the network size does not necessarily reflect the density, quality or quantity of information/resources available; however, these limitations are accepted due to data availability restraints (Knight & Yueh, 2008; Peng et al., 2021). It is assumed that as the network has migration experience, the probability of them being “redundant” relationships is mitigated. An absence of guidance regarding a network size “threshold” resulted in the threshold being constructed relatively. The mean number of family/friends outside the household is 5, the median is 4 and the mode is 3. Based on these summary statistics, a household is said to be deprived in social networks if the total number is below 3, which is also below the sample median. Access to training or NGO/governmental support, defined as a social capital measure by Gannon & Roberts (2020), could not be included due to high missing data.

Physical (roof material, latrine type, drinking water, overcrowding, housing tenure)

Inadequate roofing is defined as one made of natural materials (Gordon, 2005) or one that lacks cement, slabs or tiling (Catalan, 2017). Within the DECCMA survey, a household was not deprived if the roof material was stone/brick/slate or cement/tiles/asbestos, whereas the household was recorded as deprived if the roof was constructed from tin/corrugate, hay/leaves/branches or “other”.

WHO (2022a) defines improved latrine sanitation as private facilities that “separate human excreta from human contact”. Respondents who recorded having either a flushing latrine, a pit latrine or a ventilated pit (KVIP) were deemed to have improved facilities; whereas those without a facility or primarily using a public toilet were recorded as deprived.

WHO (2022b) defines an improved drinking water source as “piped water, public tap, borehole or pump, protected well or protected spring”. Based on the categories available in the DECCMA survey, households with piped water, standpipe or tubewell/borehole were defined as having an improved source. Contrastingly, those relying on dug wells, springs, rainwater or surface water were deemed to be deprived. 199 households stated they used an “other” drinking water source that was not listed. 84% of these households stated that they were either moderately or very happy with their drinking water; therefore, it was assumed those that stated “other” were not deprived. For reference, a comparative 83% households with piped/standpipe/borehole water were moderately/very happy with their drinking water.

Overcrowding was incorporated within the GSS multiple deprivation study, defined as having 3+ people per room in a dwelling (GSS, 2020). However, the DECCMA dataset did not possess information on the number of rooms in the household. Therefore, to evaluate overcrowding, house size (m^2^) was divided by the number of people in the household, to give a result of people per m^2^ house space. A UN study in 2000 of 9 African countries stated the average floor space per person was 5-9m^2^ (Ramalhete et al., 2018). A threshold for overcrowding was taken at 5m^2^ and 9m^2^ to test the measure's sensitivity. If using a 5m^2^ threshold then only 2.7% sample would be deemed “overcrowded”; this is too restrictive and could be criticised for its lack of “specificity” (Rose et al., 2013). Contrastingly, when using a threshold of 9m^2^, 16% households were recorded as overcrowded. Selecting the 9m^2^ threshold ensures that those defined as overcrowded have less floor space than the average household in the sampled African countries (Ramalhete et al., 2018).

The final physical capital indicator reflects home ownership and tenure security. Many multidimensional wellbeing measures emphasise the importance of secure tenure and ownership as a strategy to increase productivity, safety, remove poverty, “free-up” capital for productive investments (Adarkwa, 2010), reduce health issues emerging from the uncertainty of having an “exclusive” place to call home (Luginaah et al., 2010), and ensure universally recognised basic living standards are met (Alkire & Santos, 2010; El-hadj et al., 2018; Lemanski, 2011; Santos & Villatoro, 2018). Households that outright owned their property were defined as non-deprived, whereas households that either rented, mortgaged or squatted in their property were deemed to be deprived. Due to high rent/mortgage costs in urban areas, and preference for self-construction in poorer, rural areas (Cobbinah & Niminga-Beka, 2017; Decardi-Nelson et al., 2012; Parby et al., 2015), it is hypothesised that housing tenure will be less prevalent in more-affluent, peri-urban areas. However, “housing tenure” is not incorporated to reflect monetary wealth, it is an “end” of deprivation in itself.

**Appendix C** Descriptive sociodemographic statistics for the DECCMA household survey sample

| **Sociodemographic characteristic** | **Descriptive statistics** |
| --- | --- |
| Region | Volta – 878 (64%)  Greater Accra – 486 (36%) |
| Household size | 1-person – 242 (18%)  2-3 person – 401 (29%)  4-5 person – 409 (30%)  6-7 person – 204 (15%)  8+ person – 108 (8%) |
| Number of dependants in household | 0 – 331 (24%)  1-2 – 553 (41%)  3-4 – 349 (26%)  5+ – 131 (10%) |
| How long have you lived in the village? | Entire life – 732 (54%)  Migrated 10+ year ago – 360 (26%)  Migrated <10 years ago – 265 (19%)  *Missing – 7 (1%)* |
| Household member currently migrated | Yes – 603 (44%)  No – 761 (56%) |
| Religion | Christian – 979 (72%)  Traditional – 305 (22%)  Other (inc. Islam & Buddhism) – 77 (6%)  *Missing – 3 (<1%)* |
| Household head education | No schooling – 382 (28%)  Primary & Lower Secondary (below basic) – 391 (29%)  Upper Secondary (basic education) – 338 (25%)  Higher education – 249 (18%)  *Missing – 4 (<1%)* |
| Household head marital status | Married or Cohabitating – 815 (60%)  Never married – 158 (12%)  Previously married (divorced or widowed) – 385 (28%)  *Missing – 4 (<1%)* |
| Household head employment status | Permanent – 937 (69%)  Non-Permanent (i.e., seasonal) – 281 (21%)  Dependant (i.e., student or retiree) – 125 (9%)  *Missing – 21 (2%)* |
| Household head sex | Male – 802 (59%)  Female – 558 (41%)  *Missing – 4 (<1%)* |
| Household head age | 18-33 years – 344 (25%)  34-45 years – 350 (26%)  46-60 years – 345 (25%)  60+ years – 321 (24%)  *Missing – 4 (<1%)* |

**Appendix D** Defining characteristics (wellbeing, landscape, livelihoods) used to justify the selection of the eight study sites. Defining characteristics were based on statistical analysis of the DECCMA dataset which identified communities with above/below-average wellbeing outcomes, livelihood types and landscape properties

|  | **Key characteristics used to justify selection** | | |
| --- | --- | --- | --- |
| **Site** | **Wellbeing outcomes** | **Landscape** | **Livelihoods** |
| **1)**  **Afienya** | - Lower expenditure poverty - Lower basic needs deprivation | - Proximity to Tema/Accra - Inland | - Non-primary (i.e., mechanics) |
| **2) Anyamam** | - Higher basic needs deprivation, financial stress and unhappiness. - Higher odds of opposing “non-poor/unhappy” | - Near marshland - Near lagoon & coast | - Fishing - Salt mining |
| **3) Sogakope** | - Higher proportion “non-poor/unhappy” - Higher financial stress | - Within district capital - Proximity to river - Wetland and river vegetation coverage | - Non-primary (i.e., tourism & construction) |
| **4) Nyitawuta** | - Higher expenditure poverty - Lower financial stress and unhappiness (“poor/happy”) | - Inland, remote village - High grassland coverage | - Agricultural |
| **5) Awlikope** | - “Poor/happy” outcomes | - High cropland coverage | - Agricultural - NGO support |
| **6)**  **Aflao** | - Lower basic needs deprivation | - High built-up coverage - Close to Togo border - High accessibility to roads - Near coast | - Fishing - Nearby non-primary factory opportunities |
| **7)**  **Kedzi** | - Higher “non-poor/happy” | - Near lagoon & coast - Located on reclaimed spit following loss of old town to the ocean. | - Fishing |
| **8)**  **Anloga** | - Higher “non-poor/happy” - Low financial stress, contrasting nearby village with 100% | - Near coast, river & lagoon - Wetland coverage | - Fishing - Farming |

**Appendix E** Details of focus group and interview participants from all eight study sites. Age profiles for all community interviews, focus group members, and DPOs also presented. Note, some individuals did not disclose their age, also some individuals who consented left during FGs. However, as it is impossible to know which individual departed, their ages are included in the figures.

| **1) Afienya** | Male FG | Female FG | Community interviews | DPO interview |
| --- | --- | --- | --- | --- |
| No. participants | 7 | 6 | 1 (M) | 1 (F) |
| Duration | 01:45 | 01:38 | 00:33 | 00:55 |
| Age range | 32 - 49 | 21 - 55 | 32 – 41 | 42 - 51 |
| Language | English | Dangbe | Dangbe | English |
| Consent | Written | Written | Written | Written |

| **2) Anyamam** | Male FG | Female FG | Community interviews | DPO interview |
| --- | --- | --- | --- | --- |
| No. participants | 8 | 8 | 1 (M), 1 (F) | 1 (F) |
| Duration | 01:47 | 02:09 | 00:42 (M), 00:49 (F) | 00:59 |
| Age range | 28 - 48 | 25 - 62 | 32 – 41 (M & F) | 32 - 41 |
| Language | Dangbe | Dangbe | Dangbe | English |
| Consent | Written | Written | Written | Written |

| **3) Sogakope** | Male FG | Female FG | Community interviews | DPO interview |
| --- | --- | --- | --- | --- |
| No. participants | 6 | 10 | 1 (M), 1 (F) | 1 (M) |
| Duration | 01:32 | 01:49 | 00:32 (M), 00:32 (F) | 00:46 |
| Age range | 30 - 57 | 34 - 55 | 26 – 31 (M), 18 – 25 (F) | 32 - 41 |
| Language | Ewe | Ewe | English (M & F) | English |
| Consent | Written | Written | Written | Written |

| **4) Nyitawuta** | Male FG | Female FG | Community interviews | DPO interview |
| --- | --- | --- | --- | --- |
| No. participants | 8 | 8 | 1 (M), 1 (F) | 1 (M) |
| Duration | 02:08 | 01:42 | 00:46 (M), 00:47 (F) | 00:36 |
| Age range | 20 - 80 | 36 - 63 | x (M), 42 – 51 (F) | x |
| Language | Ewe | Ewe | Ewe | English |
| Consent | Written | Written | Written | Spoken |

| **5) Awlikope** | Male FG | Female FG | Community interviews | DPO interview |
| --- | --- | --- | --- | --- |
| No. participants | 8 | 8 | 1 (M), 1 (F) | 1 (M) |
| Duration | 01:51 | 01:52 | 00:58 (M), 01:03 (F) | 00:41 |
| Age range | 35 - 50 | 26 - 62 | 62 – 71 (M), 32 – 41 (F) | 42 - 51 |
| Language | Ewe | Ewe | English (M), Ewe (F) | English |
| Consent | Written | Written | Written | Written |

| **6) Aflao** | Male FG | Female FG | Community interviews | DPO interview |
| --- | --- | --- | --- | --- |
| No. participants | 7 | 8 | 1 (M), 1 (F) | 1 (M) |
| Duration | 01:04 | 01:27 | 00:37 (M), 00:39 (F) | 00:47 |
| Age range | 26 - 60 | 19 - 63 | 32 – 41 (M), 62 – 71 (F) | x |
| Language | Ewe | Ewe | Ewe (M), Ewe/Dangbe (F) | English |
| Consent | Written | Written | Written | Spoken |

| **7) Kedzi** | Male FG | Female FG | Community interviews | DPO interview |
| --- | --- | --- | --- | --- |
| No. participants | 9 | 7 | 2 (M) | 1 (M) |
| Duration | 01:51 | 01:45 | 00:45, 01:02 (M) | 00:38 |
| Age range | 32 - 66 | 38 - 58 | 32 – 41, 42 – 51 (M) | 52 - 61 |
| Language | Ewe | Ewe | 2 English (M) | English |
| Consent | Written | Written | Written | Written |

| **8) Anloga** | Male FG | Female FG | Community interviews | DPO interview |
| --- | --- | --- | --- | --- |
| No. participants | 10 | 8 | 1 (M), 1 (F) | 1 (M) |
| Duration | 01:30 | 01:40 | 00:48 (M), 00:37 (F) | 00:46 |
| Age range | 28 - 63 | 18 - 59 | 32 – 41 (M & F) | 32 – 41 |
| Language | Ewe | Ewe | English (M), Ewe (F) | English |
| Consent | Written | Written | Written | Written |

**Appendix F** Phi binary correlation coefficients for each combination of the 12 deprivation indicators. Only 5 combinations have a strong association according to Akoglu’s (2018) thresholds; >0 none/very weak, >0.05 weak, >0.10 moderate, >0.15 strong, >0.25 very strong. None of the “strong” associations occur within the same basic need capital category.

|  |  | Financial | | | Human | | Social | | Physical | | | | |
| --- | --- | --- | --- | --- | --- | --- | --- | --- | --- | --- | --- | --- | --- |
|  | Deprivation | Employment | Excess capital | Bank access | Education | Healthcare access | Cooperative membership | Network size | Roof material | Safe latrine | Drinking source | Overcrowding | Home ownership |
| Financial | Employment |  | 0.12 | 0.05 | 0.00 | 0.10 | 0.04 | 0.01 | 0.00 | 0.08 | 0.05 | 0.02 | 0.03 |
|  | Excess capital |  |  | 0.05 | 0.12 | 0.02 | 0.05 | 0.01 | 0.01 | 0.08 | 0.01 | 0.01 | 0.01 |
|  | Bank access |  |  |  | 0.19 | 0.05 | 0.10 | 0.02 | 0.17 | 0.04 | 0.11 | 0.00 | 0.10 |
| Human | Education |  |  |  |  | 0.04 | 0.07 | 0.13 | 0.03 | 0.16 | 0.05 | 0.11 | 0.05 |
|  | Healthcare access |  |  |  |  |  | 0.10 | 0.10 | 0.09 | 0.28 | 0.01 | 0.04 | 0.13 |
| Social | Cooperative membership |  |  |  |  |  |  | 0.08 | 0.02 | 0.10 | 0.07 | 0.01 | 0.02 |
|  | Network size |  |  |  |  |  |  |  | 0.07 | 0.16 | 0.00 | 0.11 | 0.05 |
| Physical | Roof quality |  |  |  |  |  |  |  |  | 0.08 | 0.11 | 0.03 | 0.07 |
|  | Safe latrine |  |  |  |  |  |  |  |  |  | 0.05 | 0.03 | 0.11 |
|  | Drinking source |  |  |  |  |  |  |  |  |  |  | 0.05 | 0.07 |
|  | Overcrowding |  |  |  |  |  |  |  |  |  |  |  | 0.02 |
|  | Homeownership |  |  |  |  |  |  |  |  |  |  |  |  |

**Appendix G** Supporting pictograms for the 12 basic needs components weighted by community focus groups and district planning officers.

| **Capital** | **Indicator** | **Pictogram** |
| --- | --- | --- |
| Financial | Employment | 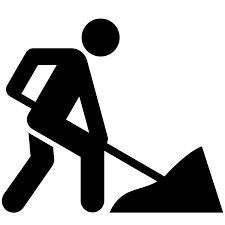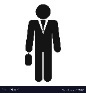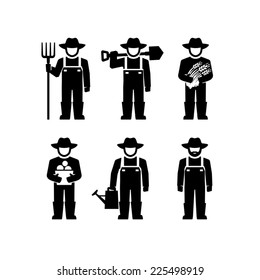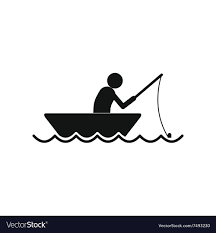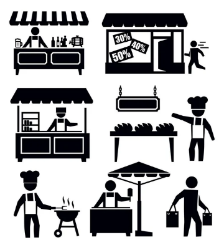 |
|  | Excess capital (% food expenditure) | 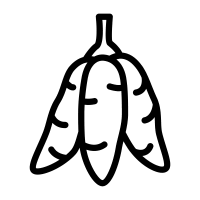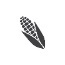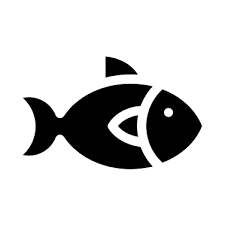 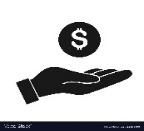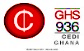 |
|  | Bank access | 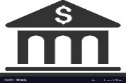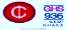 |
| Human | Education | 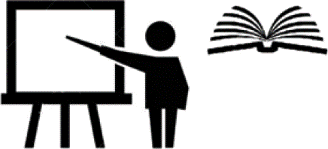 |
|  | Healthcare access | 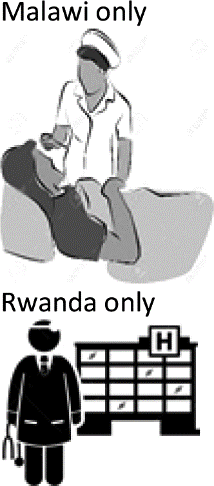 |
| Social | Cooperative membership | 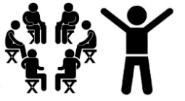 |
|  | Family/friends network size | 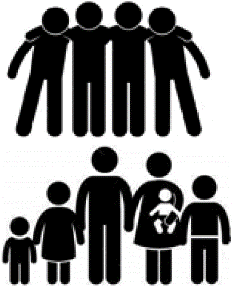 |

| **Capital** | **Indicator** | **Pictogram** |
| --- | --- | --- |
| Physical | Roof material | 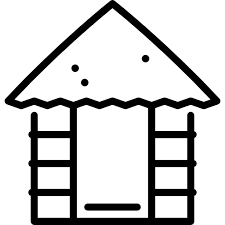 |
|  | Latrine facility | 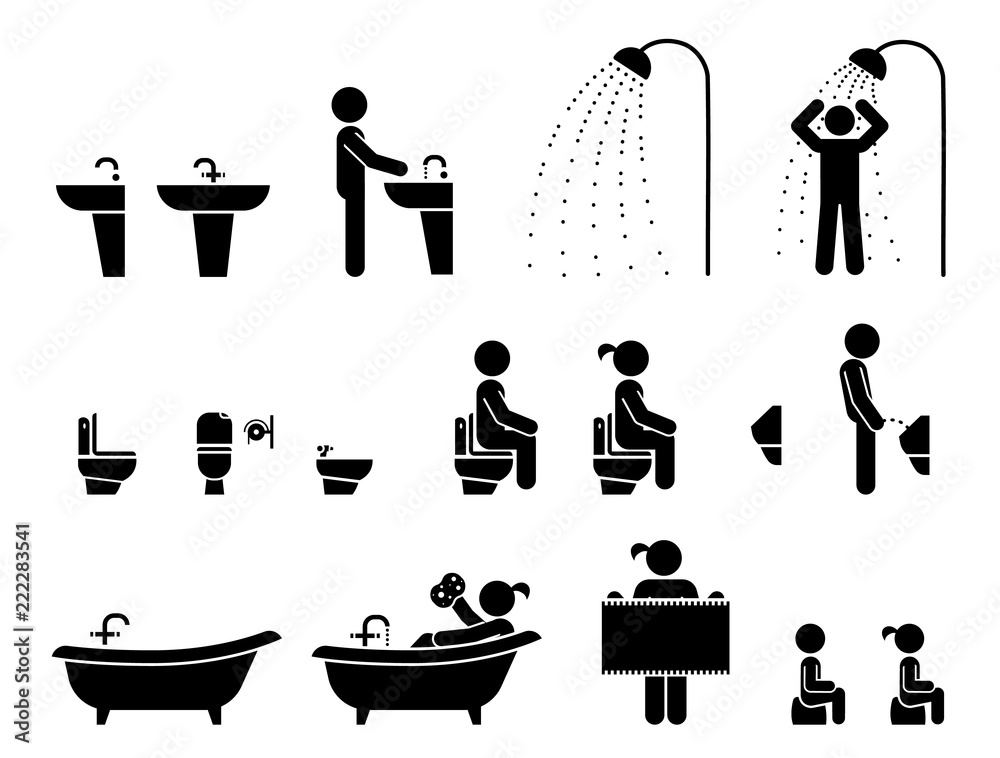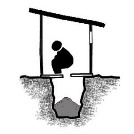 |
|  | Drinking water | 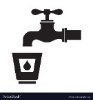 |
|  | No crowding | 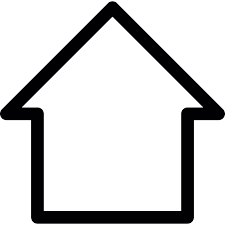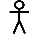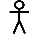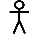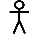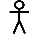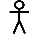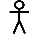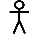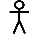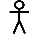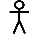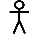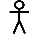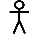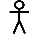 |
|  | Home ownership | 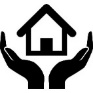 |

**Appendix H** Individual scores provided during community focus groups and DPO interviews. Summed community-level weights are presented below the individual-level scores. Summed community-level weights are compared to the baseline “nested” weights. The highest community weight for each deprivation is highlighted grey.

**Individual scores:**

|  |  |  | **Male respondents** | | | | | | | | | | | **Female respondents** | | | | | | | | | | |
| --- | --- | --- | --- | --- | --- | --- | --- | --- | --- | --- | --- | --- | --- | --- | --- | --- | --- | --- | --- | --- | --- | --- | --- | --- |
| **Location** | **Weighting round** | **Indicator** | **1** | **2** | **3** | **4** | **5** | **6** | **7** | **8** | **9** | **10** | **DPO** | **1** | **2** | **3** | **4** | **5** | **6** | **7** | **8** | **9** | **10** | **DPO** |
| 1 - Afienya | 1 | Employment | 4 | 3 | 6 | 5 | 5 | 3 | 6 | x | x | x | x | 4 | 5 | 5 | 5 | 5 | 5 | x | x | x | x | 5 |
|  |  | Excess capital | 4 | 7 | 3 | 3 | 5 | 5 | 3 | x | x | x | x | 3 | 2 | 3 | 0 | 2 | 3 | x | x | x | x | 3 |
|  |  | Bank access | 2 | 0 | 1 | 2 | 0 | 2 | 1 | x | x | x | x | 3 | 3 | 2 | 5 | 3 | 2 | x | x | x | x | 2 |
|  | 2 | Education | 5 | 4 | 4 | 4 | 5 | 6 | 5 | x | x | x | x | 3 | 4 | 3 | 5 | 4 | 3 | x | x | x | x | 5 |
|  |  | Healthcare access | 5 | 6 | 6 | 6 | 5 | 4 | 5 | x | x | x | x | 7 | 6 | 7 | 5 | 6 | 7 | x | x | x | x | 5 |
|  | 3 | Cooperative | 4 | 2 | 3 | 3 | 5 | 2 | 4 | x | x | x | x | 10 | 8 | 10 | 8 | 8 | 0 | x | x | x | x | 5 |
|  |  | Network size | 6 | 8 | 7 | 7 | 5 | 8 | 6 | x | x | x | x | 0 | 2 | 0 | 2 | 2 | 10 | x | x | x | x | 5 |
|  | 4 | Roof | 3 | 0 | 2 | 2 | 1 | 0 | 1 | x | x | x | x | 0 | 0 | 0 | 0 | 0 | 0 | x | x | x | x | 0 |
|  |  | Drinking water | 3 | 2 | 1 | 4 | 3 | 2 | 2 | x | x | x | x | 2 | 2 | 2 | 1 | 2 | 2 | x | x | x | x | 3 |
|  |  | Toilet | 1 | 0 | 0 | 2 | 1 | 3 | 1 | x | x | x | x | 2 | 2 | 2 | 2 | 1 | 2 | x | x | x | x | 3 |
|  |  | No crowding | 0 | 3 | 5 | 0 | 0 | 2 | 3 | x | x | x | x | 4 | 4 | 4 | 5 | 5 | 4 | x | x | x | x | 0 |
|  |  | Home ownership | 3 | 5 | 2 | 2 | 5 | 3 | 3 | x | x | x | x | 2 | 2 | 2 | 2 | 2 | 2 | x | x | x | x | 4 |
|  | 5 | Financial | 4 | 7 | 4 | 4 | 5 | 4 | 4 | x | x | x | x | 4 | 4 | 3 | 3 | 3 | 4 | x | x | x | x | 2 |
|  |  | Human | 3 | 0 | 3 | 3 | 2 | 2 | 3 | x | x | x | x | 3 | 3 | 4 | 5 | 4 | 2 | x | x | x | x | 3 |
|  |  | Social | 0 | 0 | 2 | 0 | 1 | 2 | 2 | x | x | x | x | 0 | 1 | 0 | 0 | 0 | 0 | x | x | x | x | 2 |
|  |  | Physical | 3 | 3 | 1 | 3 | 2 | 2 | 1 | x | x | x | x | 3 | 2 | 3 | 2 | 3 | 4 | x | x | x | x | 3 |

|  |  |  | **Male respondents** | | | | | | | | | | | **Female respondents** | | | | | | | | | | |
| --- | --- | --- | --- | --- | --- | --- | --- | --- | --- | --- | --- | --- | --- | --- | --- | --- | --- | --- | --- | --- | --- | --- | --- | --- |
| **Location** | **Weighting round** | **Indicator** | **1** | **2** | **3** | **4** | **5** | **6** | **7** | **8** | **9** | **10** | **DPO** | **1** | **2** | **3** | **4** | **5** | **6** | **7** | **8** | **9** | **10** | **DPO** |
| 2- Anyamam | 1 | Employment | 5 | 6 | 4 | 5 | 6 | 6 | 7 | 6 | x | x | x | 7 | 6 | 5 | 6 | 6 | 5 | 5 | 5 | x | x | 7 |
|  |  | Excess capital | 2 | 1 | 3 | 2 | 2 | 1 | 1 | 1 | x | x | x | 2 | 2 | 3 | 2 | 2 | 2 | 2 | 3 | x | x | 2 |
|  |  | Bank access | 3 | 3 | 3 | 3 | 2 | 3 | 2 | 3 | x | x | x | 1 | 2 | 2 | 2 | 2 | 3 | 3 | 2 | x | x | 1 |
|  | 2 | Education | 4 | 4 | 8 | 5 | 5 | 8 | 7 | 6 | x | x | x | 5 | 7 | 6 | 5 | 4 | 7 | 5 | 5 | x | x | 6 |
|  |  | Healthcare access | 6 | 6 | 2 | 5 | 5 | 2 | 3 | 4 | x | x | x | 5 | 3 | 4 | 5 | 6 | 3 | 5 | 5 | x | x | 4 |
|  | 3 | Cooperative | 4 | 2 | 4 | 4 | 4 | 3 | 4 | 3 | x | x | x | 4 | 9 | 6 | 5 | 8 | 5 | 5 | 7 | x | x | 7 |
|  |  | Network size | 6 | 8 | 6 | 6 | 6 | 7 | 6 | 7 | x | x | x | 6 | 1 | 4 | 5 | 2 | 5 | 5 | 3 | x | x | 3 |
|  | 4 | Roof | 2 | 2 | 2 | 2 | 2 | 1 | 1 | 2 | x | x | x | 2 | 1 | 1 | 1 | 1 | 1 | 1 | 1 | x | x | 1 |
|  |  | Drinking water | 3 | 3 | 1 | 2 | 2 | 3 | 3 | 3 | x | x | x | 2 | 1 | 2 | 1 | 2 | 2 | 1 | 2 | x | x | 3 |
|  |  | Toilet | 1 | 2 | 1 | 2 | 1 | 2 | 1 | 2 | x | x | x | 1 | 1 | 1 | 3 | 1 | 1 | 3 | 1 | x | x | 1 |
|  |  | No crowding | 0 | 0 | 1 | 1 | 1 | 0 | 1 | 1 | x | x | x | 1 | 2 | 2 | 1 | 1 | 1 | 3 | 1 | x | x | 1 |
|  |  | Home ownership | 4 | 3 | 5 | 3 | 4 | 4 | 4 | 2 | x | x | x | 4 | 5 | 4 | 4 | 5 | 5 | 2 | 5 | x | x | 4 |
|  | 5 | Financial | 4 | 4 | 8 | 4 | 4 | 7 | 9 | 4 | x | x | x | 4 | 5 | 4 | 4 | 2 | 2 | 6 | 4 | x | x | 4 |
|  |  | Human | 2 | 3 | 1 | 2 | 2 | 3 | 1 | 4 | x | x | x | 3 | 2 | 2 | 3 | 3 | 3 | 0 | 2 | x | x | 3 |
|  |  | Social | 1 | 0 | 0 | 2 | 2 | 0 | 0 | 1 | x | x | x | 1 | 1 | 2 | 1 | 2 | 2 | 4 | 1 | x | x | 1 |
|  |  | Physical | 3 | 3 | 1 | 2 | 2 | 0 | 0 | 1 | x | x | x | 2 | 2 | 2 | 2 | 3 | 3 | 0 | 3 | x | x | 2 |

|  |  |  | **Male respondents** | | | | | | | | | | | **Female respondents** | | | | | | | | | | |
| --- | --- | --- | --- | --- | --- | --- | --- | --- | --- | --- | --- | --- | --- | --- | --- | --- | --- | --- | --- | --- | --- | --- | --- | --- |
| **Location** | **Weighting round** | **Indicator** | **1** | **2** | **3** | **4** | **5** | **6** | **7** | **8** | **9** | **10** | **DPO** | **1** | **2** | **3** | **4** | **5** | **6** | **7** | **8** | **9** | **10** | **DPO** |
| 3 - Sogakope | 1 | Employment | 6 | 9 | 7 | 10 | 7 | 9 | x | x | x | x | 5 | 3 | 6 | 5 | 5 | 5 | 5 | 7 | 8 | 7 | 5 | x |
|  |  | Excess capital | 4 | 1 | 3 | 0 | 2 | 1 | x | x | x | x | 3 | 4 | 3 | 3 | 4 | 3 | 3 | 2 | 1 | 1 | 3 | x |
|  |  | Bank access | 0 | 0 | 0 | 0 | 1 | 0 | x | x | x | x | 2 | 3 | 1 | 2 | 1 | 2 | 2 | 1 | 1 | 2 | 2 | x |
|  | 2 | Education | 3 | 2 | 2 | 2 | 3 | 5 | x | x | x | x | 4 | 7 | 6 | 6 | 2 | 5 | 5 | 5 | 3 | 3 | 4 | x |
|  |  | Healthcare access | 7 | 8 | 8 | 8 | 7 | 5 | x | x | x | x | 6 | 3 | 4 | 4 | 8 | 5 | 5 | 5 | 7 | 7 | 6 | x |
|  | 3 | Cooperative | 3 | 6 | 2 | 5 | 7 | 4 | x | x | x | x | 3 | 0 | 3 | 0 | 2 | 0 | 0 | 2 | 2 | 0 | 4 | x |
|  |  | Network size | 7 | 4 | 8 | 5 | 3 | 6 | x | x | x | x | 7 | 10 | 7 | 10 | 8 | 10 | 10 | 8 | 8 | 10 | 6 | x |
|  | 4 | Roof | 0 | 0 | 3 | 0 | 2 | 0 | x | x | x | x | 2 | 2 | 2 | 0 | 0 | 0 | 0 | 2 | 2 | 0 | 0 | x |
|  |  | Drinking water | 0 | 2 | 2 | 2 | 2 | 2 | x | x | x | x | 3 | 2 | 2 | 2 | 2 | 2 | 2 | 2 | 4 | 2 | 0 | x |
|  |  | Toilet | 0 | 1 | 3 | 2 | 2 | 1 | x | x | x | x | 1 | 2 | 2 | 2 | 0 | 2 | 2 | 2 | 0 | 2 | 2 | x |
|  |  | No crowding | 0 | 2 | 0 | 0 | 1 | 2 | x | x | x | x | 1 | 0 | 0 | 2 | 0 | 0 | 0 | 2 | 2 | 0 | 2 | x |
|  |  | Home ownership | 10 | 5 | 2 | 6 | 3 | 5 | x | x | x | x | 3 | 4 | 4 | 4 | 8 | 6 | 6 | 2 | 2 | 6 | 6 | x |
|  | 5 | Financial | 7 | 2 | 4 | 0 | 1 | 2 | x | x | x | x | 3 | 2 | 2 | 2 | 2 | 2 | 2 | 2 | 1 | 3 | 2 | x |
|  |  | Human | 3 | 2 | 2 | 7 | 7 | 6 | x | x | x | x | 2 | 2 | 3 | 4 | 5 | 3 | 3 | 4 | 6 | 4 | 4 | x |
|  |  | Social | 0 | 3 | 2 | 0 | 1 | 0 | x | x | x | x | 2 | 2 | 2 | 3 | 2 | 3 | 3 | 2 | 2 | 2 | 2 | x |
|  |  | Physical | 0 | 3 | 2 | 3 | 1 | 2 | x | x | x | x | 3 | 4 | 3 | 1 | 1 | 2 | 2 | 2 | 1 | 1 | 2 | x |

|  |  |  | **Male respondents** | | | | | | | | | | | **Female respondents** | | | | | | | | | | |
| --- | --- | --- | --- | --- | --- | --- | --- | --- | --- | --- | --- | --- | --- | --- | --- | --- | --- | --- | --- | --- | --- | --- | --- | --- |
| **Location** | **Weighting round** | **Indicator** | **1** | **2** | **3** | **4** | **5** | **6** | **7** | **8** | **9** | **10** | **DPO** | **1** | **2** | **3** | **4** | **5** | **6** | **7** | **8** | **9** | **10** | **DPO** |
| 4 - Nyitawuta | 1 | Employment | 6 | 5 | 5 | 5 | 5 | 6 | 7 | 6 | x | x | 5 | 6 | 4 | 4 | 5 | 6 | 5 | 6 | 5 | x | x | x |
|  |  | Excess capital | 2 | 0 | 1 | 2 | 2 | 1 | 1 | 0 | x | x | 3 | 2 | 3 | 3 | 3 | 2 | 2 | 2 | 3 | x | x | x |
|  |  | Bank access | 2 | 5 | 4 | 3 | 3 | 3 | 2 | 4 | x | x | 2 | 2 | 3 | 3 | 2 | 2 | 3 | 2 | 2 | x | x | x |
|  | 2 | Education | 4 | 5 | 6 | 5 | 6 | 6 | 4 | 4 | x | x | 4 | 4 | 5 | 6 | 5 | 6 | 5 | 5 | 6 | x | x | x |
|  |  | Healthcare access | 6 | 5 | 4 | 5 | 4 | 4 | 6 | 6 | x | x | 6 | 6 | 5 | 4 | 5 | 4 | 5 | 5 | 4 | x | x | x |
|  | 3 | Cooperative | 4 | 5 | 4 | 4 | 2 | 5 | 6 | 2 | x | x | 6 | 6 | 5 | 5 | 5 | 4 | 5 | 4 | 6 | x | x | x |
|  |  | Network size | 6 | 5 | 6 | 6 | 8 | 5 | 4 | 8 | x | x | 4 | 4 | 5 | 5 | 5 | 6 | 5 | 6 | 4 | x | x | x |
|  | 4 | Roof | 2 | 1 | 1 | 1 | 1 | 1 | 0 | 0 | x | x | 2 | 1 | 3 | 2 | 2 | 3 | 2 | 2 | 2 | x | x | x |
|  |  | Drinking water | 2 | 2 | 2 | 2 | 1 | 1 | 2 | 1 | x | x | 4 | 2 | 1 | 2 | 2 | 2 | 4 | 2 | 2 | x | x | x |
|  |  | Toilet | 1 | 1 | 2 | 1 | 1 | 1 | 1 | 1 | x | x | 2 | 2 | 1 | 2 | 2 | 1 | 1 | 2 | 2 | x | x | x |
|  |  | No crowding | 4 | 2 | 4 | 4 | 4 | 5 | 3 | 4 | x | x | 1 | 3 | 3 | 3 | 2 | 3 | 1 | 1 | 2 | x | x | x |
|  |  | Home ownership | 1 | 4 | 1 | 2 | 3 | 2 | 4 | 4 | x | x | 1 | 2 | 2 | 1 | 2 | 1 | 2 | 3 | 2 | x | x | x |
|  | 5 | Financial | 4 | 5 | 5 | 4 | 5 | 6 | 4 | 7 | x | x | 3 | 4 | 4 | 4 | 4 | 3 | 4 | 3 | 3 | x | x | x |
|  |  | Human | 2 | 3 | 2 | 4 | 2 | 2 | 2 | 1 | x | x | 4 | 2 | 3 | 1 | 4 | 4 | 2 | 3 | 2 | x | x | x |
|  |  | Social | 2 | 0 | 2 | 0 | 2 | 1 | 1 | 1 | x | x | 1 | 2 | 1 | 2 | 1 | 2 | 2 | 2 | 3 | x | x | x |
|  |  | Physical | 2 | 2 | 1 | 2 | 1 | 1 | 3 | 1 | x | x | 2 | 2 | 2 | 3 | 1 | 1 | 2 | 2 | 2 | x | x | x |

|  |  |  | **Male respondents** | | | | | | | | | | | **Female respondents** | | | | | | | | | | |
| --- | --- | --- | --- | --- | --- | --- | --- | --- | --- | --- | --- | --- | --- | --- | --- | --- | --- | --- | --- | --- | --- | --- | --- | --- |
| **Location** | **Weighting round** | **Indicator** | **1** | **2** | **3** | **4** | **5** | **6** | **7** | **8** | **9** | **10** | **DPO** | **1** | **2** | **3** | **4** | **5** | **6** | **7** | **8** | **9** | **10** | **DPO** |
| 5 - Awlikope | 1 | Employment | 6 | 7 | 5 | 6 | 4 | 6 | 4 | 4 | x | x | 5 | 6 | 6 | 5 | 2 | 5 | 4 | 5 | 3 | x | x | x |
|  |  | Excess capital | 2 | 2 | 4 | 2 | 3 | 3 | 4 | 4 | x | x | 4 | 2 | 2 | 2 | 2 | 2 | 3 | 2 | 2 | x | x | x |
|  |  | Bank access | 2 | 1 | 1 | 2 | 3 | 1 | 2 | 2 | x | x | 1 | 2 | 2 | 3 | 6 | 3 | 3 | 3 | 5 | x | x | x |
|  | 2 | Education | 7 | 3 | 5 | 5 | 5 | 2 | 4 | 4 | x | x | 4 | 4 | 6 | 4 | 7 | 7 | 5 | 5 | 7 | x | x | x |
|  |  | Healthcare access | 3 | 7 | 5 | 5 | 5 | 8 | 6 | 6 | x | x | 6 | 6 | 4 | 6 | 3 | 3 | 5 | 5 | 3 | x | x | x |
|  | 3 | Cooperative | 6 | 4 | 4 | 5 | 5 | 4 | 6 | 5 | x | x | 8 | 3 | 4 | 4 | 4 | 6 | 3 | 0 | 7 | x | x | x |
|  |  | Network size | 4 | 6 | 6 | 5 | 5 | 6 | 4 | 5 | x | x | 2 | 7 | 6 | 6 | 6 | 4 | 7 | 10 | 3 | x | x | x |
|  | 4 | Roof | 0 | 2 | 2 | 1 | 0 | 2 | 0 | 2 | x | x | 1 | 0 | 0 | 0 | 0 | 0 | 1 | 0 | 1 | x | x | x |
|  |  | Drinking water | 4 | 2 | 2 | 2 | 3 | 2 | 3 | 2 | x | x | 4 | 3 | 2 | 2 | 4 | 2 | 3 | 2 | 3 | x | x | x |
|  |  | Toilet | 4 | 1 | 2 | 2 | 3 | 2 | 3 | 2 | x | x | 3 | 1 | 2 | 1 | 3 | 2 | 2 | 2 | 3 | x | x | x |
|  |  | No crowding | 0 | 0 | 0 | 0 | 0 | 0 | 0 | 0 | x | x | 0 | 3 | 1 | 1 | 1 | 2 | 1 | 2 | 1 | x | x | x |
|  |  | Home ownership | 2 | 5 | 4 | 5 | 4 | 4 | 4 | 4 | x | x | 2 | 3 | 5 | 6 | 2 | 4 | 3 | 4 | 2 | x | x | x |
|  | 5 | Financial | 4 | 3 | 4 | 4 | 4 | 3 | 3 | 4 | x | x | 1 | 1 | 4 | 2 | 2 | 4 | 2 | 2 | 3 | x | x | x |
|  |  | Human | 3 | 4 | 3 | 3 | 2 | 3 | 3 | 3 | x | x | 4 | 3 | 2 | 2 | 3 | 3 | 2 | 2 | 3 | x | x | x |
|  |  | Social | 1 | 2 | 1 | 1 | 2 | 2 | 2 | 1 | x | x | 3 | 3 | 2 | 2 | 3 | 1 | 3 | 4 | 1 | x | x | x |
|  |  | Physical | 2 | 1 | 2 | 2 | 2 | 2 | 2 | 2 | x | x | 2 | 3 | 2 | 4 | 2 | 2 | 3 | 2 | 3 | x | x | x |

|  |  |  | **Male respondents** | | | | | | | | | | | **Female respondents** | | | | | | | | | | |
| --- | --- | --- | --- | --- | --- | --- | --- | --- | --- | --- | --- | --- | --- | --- | --- | --- | --- | --- | --- | --- | --- | --- | --- | --- |
| **Location** | **Weighting round** | **Indicator** | **1** | **2** | **3** | **4** | **5** | **6** | **7** | **8** | **9** | **10** | **DPO** | **1** | **2** | **3** | **4** | **5** | **6** | **7** | **8** | **9** | **10** | **DPO** |
| 6 - Aflao | 1 | Employment | 4 | 4 | 2 | 6 | 6 | 6 | 8 | x | x | x | 4 | 5 | 5 | 4 | 4 | 6 | 4 | 4 | 5 | x | x | x |
|  |  | Excess capital | 4 | 3 | 4 | 2 | 1 | 2 | 2 | x | x | x | 4 | 3 | 3 | 4 | 4 | 3 | 3 | 3 | 3 | x | x | x |
|  |  | Bank access | 2 | 3 | 4 | 2 | 3 | 2 | 0 | x | x | x | 2 | 2 | 2 | 2 | 2 | 1 | 3 | 3 | 2 | x | x | x |
|  | 2 | Education | 5 | 5 | 6 | 4 | 5 | 6 | 4 | x | x | x | 5 | 4 | 4 | 4 | 8 | 9 | 4 | 6 | 6 | x | x | x |
|  |  | Healthcare access | 5 | 5 | 4 | 6 | 5 | 4 | 6 | x | x | x | 5 | 6 | 6 | 6 | 2 | 1 | 6 | 4 | 4 | x | x | x |
|  | 3 | Cooperative | 2 | 4 | 7 | 2 | 1 | 4 | 2 | x | x | x | 3 | 3 | 6 | 4 | 4 | 0 | 3 | 3 | 2 | x | x | x |
|  |  | Network size | 8 | 6 | 3 | 8 | 9 | 6 | 8 | x | x | x | 7 | 7 | 4 | 6 | 6 | 10 | 7 | 7 | 8 | x | x | x |
|  | 4 | Roof | 1 | 0 | 2 | 0 | 2 | 0 | 0 | x | x | x | 1 | 1 | 2 | 2 | 1 | 0 | 2 | 0 | 0 | x | x | x |
|  |  | Drinking water | 2 | 1 | 1 | 2 | 2 | 3 | 2 | x | x | x | 3 | 2 | 2 | 3 | 4 | 1 | 2 | 2 | 0 | x | x | x |
|  |  | Toilet | 2 | 2 | 2 | 2 | 2 | 1 | 2 | x | x | x | 2 | 3 | 3 | 2 | 2 | 2 | 2 | 1 | 0 | x | x | x |
|  |  | No crowding | 2 | 1 | 2 | 3 | 2 | 2 | 4 | x | x | x | 3 | 0 | 0 | 0 | 0 | 0 | 0 | 0 | 0 | x | x | x |
|  |  | Home ownership | 3 | 6 | 3 | 3 | 2 | 4 | 2 | x | x | x | 1 | 4 | 3 | 3 | 3 | 7 | 4 | 7 | 10 | x | x | x |
|  | 5 | Financial | 4 | 5 | 2 | 5 | 5 | 2 | 3 | x | x | x | 3 | 4 | 2 | 5 | 3 | 5 | 3 | 6 | 4 | x | x | x |
|  |  | Human | 3 | 1 | 2 | 1 | 2 | 4 | 4 | x | x | x | 3 | 3 | 4 | 2 | 3 | 5 | 4 | 4 | 4 | x | x | x |
|  |  | Social | 0 | 1 | 3 | 1 | 2 | 2 | 0 | x | x | x | 1 | 2 | 2 | 1 | 0 | 0 | 2 | 0 | 1 | x | x | x |
|  |  | Physical | 3 | 3 | 3 | 3 | 1 | 2 | 3 | x | x | x | 3 | 1 | 2 | 2 | 4 | 0 | 1 | 0 | 1 | x | x | x |

|  |  |  | **Male respondents** | | | | | | | | | | | **Female respondents** | | | | | | | | | | |
| --- | --- | --- | --- | --- | --- | --- | --- | --- | --- | --- | --- | --- | --- | --- | --- | --- | --- | --- | --- | --- | --- | --- | --- | --- |
| **Location** | **Weighting round** | **Indicator** | **1** | **2** | **3** | **4** | **5** | **6** | **7** | **8** | **9** | **10** | **DPO** | **1** | **2** | **3** | **4** | **5** | **6** | **7** | **8** | **9** | **10** | **DPO** |
| 7 - Kedzi | 1 | Employment | 5 | 7 | 5 | 4 | 4 | 6 | 4 | 4 | 6 | x | 5 | 5 | 7 | 5 | 5 | 5 | 6 | 6 | x | x | x | x |
|  |  | Excess capital | 3 | 2 | 2 | 4 | 3 | 2 | 3 | 3 | 2 | x | 3 | 3 | 2 | 4 | 5 | 4 | 2 | 3 | x | x | x | x |
|  |  | Bank access | 2 | 1 | 3 | 2 | 3 | 2 | 3 | 3 | 2 | x | 2 | 2 | 1 | 1 | 0 | 1 | 2 | 1 | x | x | x | x |
|  | 2 | Education | 3 | 2 | 3 | 4 | 2 | 4 | 5 | 5 | 3 | x | 5 | 3 | 5 | 4 | 4 | 5 | 6 | 4 | x | x | x | x |
|  |  | Healthcare access | 7 | 8 | 7 | 6 | 8 | 6 | 5 | 5 | 7 | x | 5 | 7 | 5 | 6 | 6 | 5 | 4 | 6 | x | x | x | x |
|  | 3 | Cooperative | 4 | 4 | 2 | 4 | 4 | 6 | 4 | 5 | 3 | x | 6 | 2 | 3 | 2 | 3 | 1 | 2 | 4 | x | x | x | x |
|  |  | Network size | 6 | 6 | 8 | 6 | 6 | 4 | 6 | 5 | 7 | x | 4 | 8 | 7 | 8 | 7 | 9 | 8 | 6 | x | x | x | x |
|  | 4 | Roof | 0 | 0 | 0 | 0 | 0 | 0 | 0 | 0 | 0 | x | 3 | 1 | 2 | 2 | 1 | 2 | 2 | 1 | x | x | x | x |
|  |  | Drinking water | 3 | 7 | 2 | 3 | 4 | 5 | 2 | 3 | 3 | x | 3 | 1 | 2 | 2 | 3 | 2 | 3 | 2 | x | x | x | x |
|  |  | Toilet | 2 | 1 | 3 | 2 | 3 | 5 | 2 | 3 | 2 | x | 2 | 1 | 2 | 2 | 2 | 2 | 2 | 1 | x | x | x | x |
|  |  | No crowding | 1 | 1 | 1 | 2 | 3 | 0 | 2 | 0 | 1 | x | 1 | 1 | 2 | 2 | 3 | 2 | 1 | 4 | x | x | x | x |
|  |  | Home ownership | 4 | 1 | 4 | 3 | 0 | 0 | 4 | 4 | 4 | x | 1 | 6 | 2 | 2 | 1 | 2 | 2 | 2 | x | x | x | x |
|  | 5 | Financial | 2 | 1 | 2 | 3 | 3 | 2 | 3 | 3 | 2 | x | 2 | 5 | 5 | 5 | 5 | 6 | 4 | 5 | x | x | x | x |
|  |  | Human | 4 | 7 | 2 | 3 | 2 | 3 | 4 | 3 | 4 | x | 2 | 3 | 2 | 2 | 3 | 4 | 4 | 3 | x | x | x | x |
|  |  | Social | 3 | 1 | 2 | 2 | 2 | 3 | 1 | 2 | 2 | x | 4 | 1 | 1 | 1 | 1 | 0 | 1 | 1 | x | x | x | x |
|  |  | Physical | 1 | 1 | 4 | 2 | 3 | 2 | 2 | 2 | 2 | x | 2 | 1 | 2 | 2 | 1 | 0 | 1 | 1 | x | x | x | x |

|  |  |  | **Male respondents** | | | | | | | | | | | **Female respondents** | | | | | | | | | | |
| --- | --- | --- | --- | --- | --- | --- | --- | --- | --- | --- | --- | --- | --- | --- | --- | --- | --- | --- | --- | --- | --- | --- | --- | --- |
| **Location** | **Weighting round** | **Indicator** | **1** | **2** | **3** | **4** | **5** | **6** | **7** | **8** | **9** | **10** | **DPO** | **1** | **2** | **3** | **4** | **5** | **6** | **7** | **8** | **9** | **10** | **DPO** |
| 8 - Anloga | 1 | Employment | 10 | 5 | 8 | 5 | 5 | 7 | 8 | 10 | 0 | 5 | 5 | 6 | 5 | 6 | 6 | 6 | 7 | 6 | 5 | x | x | x |
|  |  | Excess capital | 0 | 3 | 2 | 2 | 2 | 3 | 2 | 0 | 10 | 3 | 5 | 2 | 3 | 2 | 3 | 2 | 2 | 2 | 4 | x | x | x |
|  |  | Bank access | 0 | 2 | 0 | 3 | 3 | 0 | 0 | 0 | 0 | 2 | 0 | 2 | 2 | 2 | 1 | 2 | 1 | 2 | 1 | x | x | x |
|  | 2 | Education | 4 | 3 | 3 | 4 | 4 | 3 | 3 | 4 | 5 | 4 | 4 | 5 | 5 | 4 | 7 | 4 | 4 | 4 | 4 | x | x | x |
|  |  | Healthcare access | 6 | 7 | 7 | 6 | 6 | 7 | 7 | 6 | 5 | 6 | 6 | 5 | 5 | 6 | 3 | 6 | 6 | 6 | 6 | x | x | x |
|  | 3 | Cooperative | 2 | 1 | 1 | 3 | 2 | 3 | 1 | 2 | 4 | 2 | 5 | 3 | 2 | 8 | 3 | 2 | 3 | 3 | 2 | x | x | x |
|  |  | Network size | 8 | 9 | 9 | 7 | 8 | 7 | 9 | 8 | 6 | 8 | 5 | 7 | 8 | 2 | 7 | 8 | 7 | 7 | 8 | x | x | x |
|  | 4 | Roof | 2 | 1 | 2 | 1 | 2 | 2 | 1 | 1 | 2 | 1 | 1 | 0 | 0 | 1 | 0 | 0 | 0 | 0 | 0 | x | x | x |
|  |  | Drinking water | 2 | 3 | 1 | 3 | 2 | 3 | 3 | 1 | 2 | 3 | 3 | 3 | 4 | 1 | 2 | 3 | 6 | 2 | 2 | x | x | x |
|  |  | Toilet | 2 | 2 | 1 | 1 | 1 | 1 | 1 | 2 | 2 | 1 | 3 | 1 | 0 | 1 | 2 | 2 | 0 | 2 | 3 | x | x | x |
|  |  | No crowding | 1 | 1 | 2 | 1 | 2 | 1 | 1 | 2 | 2 | 1 | 2 | 2 | 3 | 1 | 1 | 2 | 1 | 4 | 1 | x | x | x |
|  |  | Home ownership | 3 | 3 | 4 | 4 | 3 | 3 | 4 | 4 | 2 | 4 | 1 | 4 | 3 | 6 | 5 | 3 | 3 | 2 | 4 | x | x | x |
|  | 5 | Financial | 4 | 4 | 5 | 4 | 4 | 4 | 4 | 5 | 2 | 4 | 2 | 4 | 2 | 7 | 1 | 5 | 6 | 5 | 5 | x | x | x |
|  |  | Human | 2 | 2 | 1 | 2 | 2 | 2 | 2 | 2 | 3 | 2 | 3 | 2 | 4 | 1 | 4 | 2 | 1 | 2 | 3 | x | x | x |
|  |  | Social | 2 | 2 | 2 | 2 | 2 | 2 | 2 | 1 | 2 | 2 | 2 | 2 | 2 | 1 | 3 | 1 | 1 | 2 | 1 | x | x | x |
|  |  | Physical | 2 | 2 | 2 | 2 | 2 | 2 | 2 | 2 | 3 | 2 | 3 | 2 | 2 | 1 | 2 | 2 | 2 | 1 | 1 | x | x | x |

|  |  |  | **Community** | | | | | | | |
| --- | --- | --- | --- | --- | --- | --- | --- | --- | --- | --- |
| **Capital** | **Indicator** | **Nested baseline** | **1 –**  **Afienya** | **2 – Anyamam** | **3 –Sogakope** | **4 –**  **Nyitawuta** | **5 –Awlikope** | **6 –**  **Aflao** | **7 –**  **Kedzi** | **8 –**  **Anloga** |
| **Financial** | Employment | 1.0 | 2.3 | 3.2 | 1.8 | 2.8 | 1.8 | 2.3 | 2.2 | 2.9 |
|  | Excess capital | 1.0 | 1.6 | 1.1 | 0.6 | 0.9 | 0.9 | 1.4 | 1.2 | 1.3 |
|  | Bank access | 1.0 | 1.0 | 1.4 | 0.3 | 1.5 | 0.9 | 1.0 | 0.8 | 0.6 |
| **Human** | Education | 1.5 | 1.4 | 1.5 | 1.9 | 1.5 | 1.7 | 2.0 | 1.5 | 1.1 |
|  | Healthcare access | 1.5 | 2.0 | 1.2 | 3.0 | 1.4 | 1.7 | 1.7 | 2.4 | 1.6 |
| **Social** | Cooperative | 1.5 | 0.4 | 0.7 | 0.5 | 0.8 | 1.0 | 0.4 | 0.6 | 0.6 |
|  | Network size | 1.5 | 0.4 | 0.8 | 1.6 | 1.0 | 1.3 | 0.9 | 1.2 | 1.6 |
| **Physical** | Roof quality | 0.6 | 0.2 | 0.3 | 0.2 | 0.3 | 0.2 | 0.2 | 0.1 | 0.2 |
|  | Drinking water | 0.6 | 0.6 | 0.4 | 0.4 | 0.4 | 0.7 | 0.4 | 0.6 | 0.6 |
|  | Latrine | 0.6 | 0.4 | 0.3 | 0.4 | 0.3 | 0.6 | 0.4 | 0.4 | 0.3 |
|  | No crowding | 0.6 | 0.9 | 0.2 | 0.2 | 0.6 | 0.2 | 0.2 | 0.3 | 0.4 |
|  | Home ownership | 0.6 | 0.8 | 0.9 | 1.1 | 0.5 | 1.0 | 1.0 | 0.5 | 0.8 |

**Summed community-level weights (rounded to 1.d.p) – highest weight for each indicator highlighted:**

**Appendix I** Sensitivity analysis was undertaken to check the validity of the three-group livelihood classification. The livelihood group deprivation rates were recalculated after omitting the three surveyed communities with the greatest average distance from the visited communities of the same livelihood type.

| **Livelihood group** | **“Nested” rate** | **Community livelihood rate** | **Community livelihood rate (excluding the 3 most-distant communities of the same livelihood type)** |
| --- | --- | --- | --- |
| Farming (n=22) | 52% | 38% | 36% |
| Fishing (n=16) | 58% | 43% | 42% |
| Peri-urban (n=12) | 32% | 25% | 33% |

Similar rates were produced for farming and fishing groups, yet a slightly larger difference (8%) was noted amongst the peri-urban group. The three peri-urban communities removed were all based in Ketu South. By removing these three communities the deprivation rate increases, this is likely attributable to the high weight applied to “healthcare access” amongst the visited peri-urban sites, yet the lower level of “healthcare access” deprivation in Ketu South. Only 3 households in the omitted communities were defined as deprived overall, with none located >5km from the nearest hospital. Ketu South has four hospitals located in close proximity to one another on the main road, near the surveyed communities (Sape Agbo Memorial Hospital, New Hope Hospital, Central Aflao Hospital & Ketu South District Hospital).

The similarity of the farming and fishing rates validates the livelihood classification; however, the difference in peri-urban rates highlights the importance of local-specific research, and how different characteristics other than livelihood can influence wellbeing priorities. Compared to the visited peri-urban sites (Afienya and Sogakope), the built-up areas in Ketu South have distinct characteristics which may have resulted in the different levels of deprivation. For example, Ketu South is coastal, with four proximate hospitals, access to the main road to Tema & Accra, and located on the Togolese border – allowing traders to sell and buy products in different markets and currencies to achieve the greatest profit. The diversity of landscapes and livelihood opportunities in Ketu South may have also contributed towards the three omitted communities having a lower proportion of “employment” deprivation, further contributing towards the higher deprivation rate following their exclusion.

Classifying communities by livelihood was a pragmatic option, with livelihood strategies capable of capturing various individual and community resources; as illustrated in the Sustainable Livelihoods Framework (Scoones, 1998). All the different factors capable of influencing wellbeing priorities and objective deprivations are unable to be captured within a parsimonious, quantitative exercise. Therefore, these findings advocate for the inclusion of mixed methods when collecting community-preference weightings to enrich the collected data, and capture the context-specific challenges faced within select communities.

**Appendix J** Maps illustrating the difference in the weighted incidence when applying the corresponding community livelihood weights and DPO weights. **J.1) “**employment” deprivation **J.2)** “cooperative membership” deprivation **J.3)** “drinking water” deprivation. Household weighted incidences are calculated by multiplying each households’ binary deprivation indicator (0/1) by the corresponding weight. For example, if a household experiences “employment” deprivation, within a farming livelihood community, the weighted incidence would be (1 x 2.51). These values are summed within each community, to give an overall weighted incidence for each enumeration area.

**J.1) Employment** (Weights – DPO; 1.54, Farming; 2.51, Fishing; 2.54, Peri-urban; 2.10)


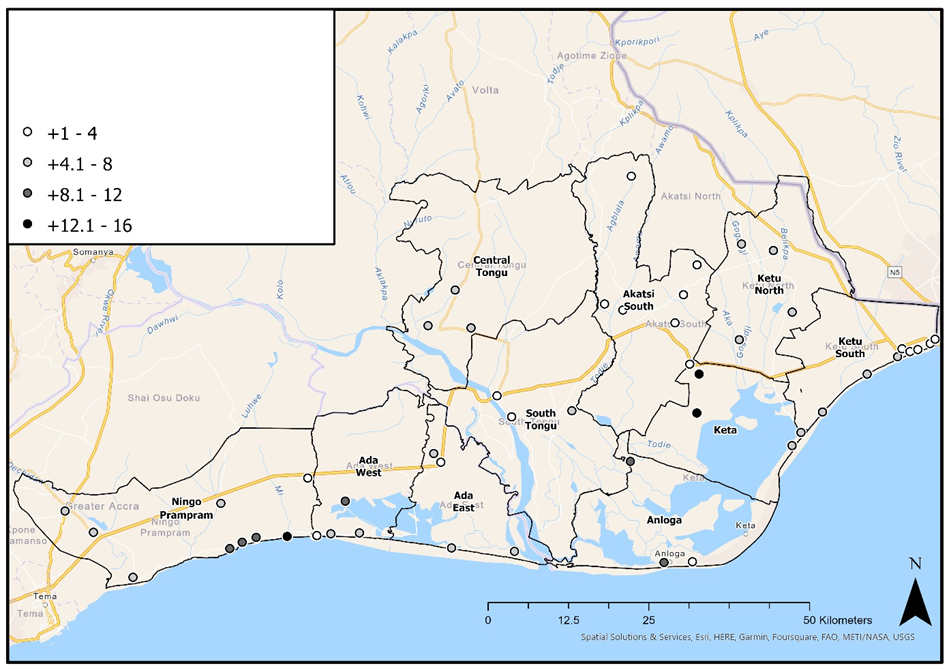


**Difference in weighted “employment” deprivation incidence**

(Community-weighted incidence minus DPO-weighted incidence)

**J.2) Cooperative membership** (Weights – DPO; 1.29, Farming; 0.80, Fishing; 0.59, Peri-urban; 0.56)

**
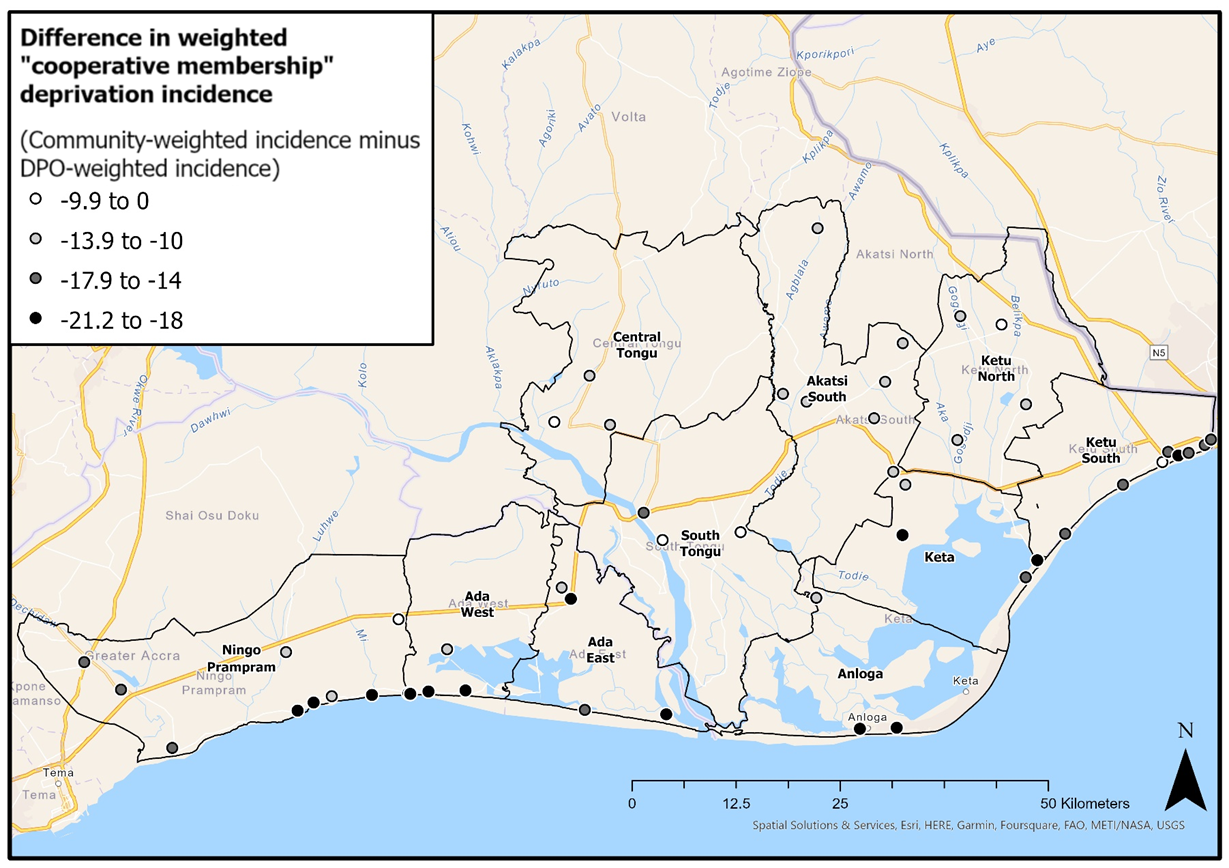
**

**J.3) Drinking water** (Weights – DPO; 0.98, Farming; 0.56, Fishing; 0.50, Peri-urban; 0.51)

**
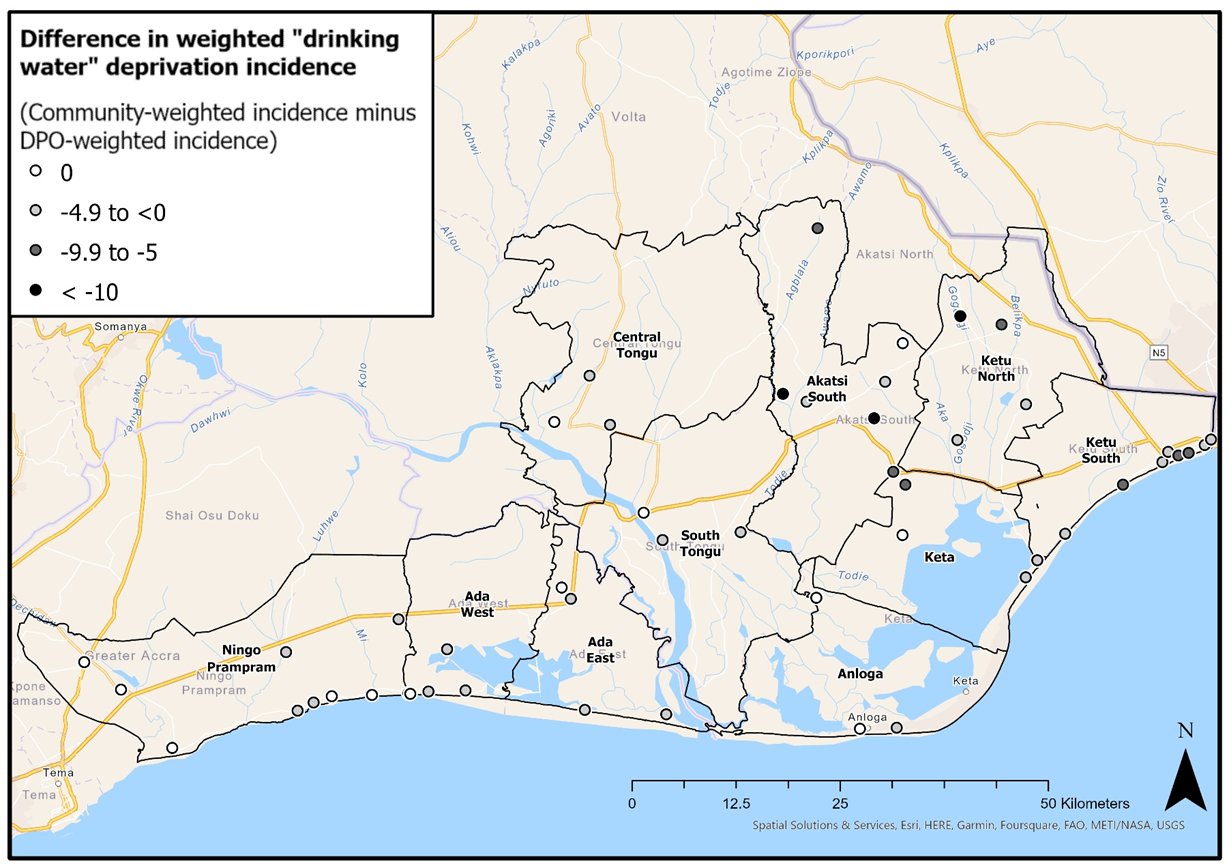
**

**Appendix K** Mean equivalised, adjusted annual household expenditure by proportion of community built-up landcover.

| **Proportion of built-up landcover in the community** | **Mean household expenditure (cedi)** | **No. households** |
| --- | --- | --- |
| None/lowest (0 – 8%) | 2678.1 | 650 |
| Low (9 - 20%) | 2986.6 | 327 |
| Medium (21 – 44%) | 4392.3 | 194 |
| High (45 – 69%) | 3528.5 | 189 |

**Appendix L** Exploratory analysis highlighting DPOs’ broader perspective of communities’ wellbeing, compared to community FG respondents.

**L.1)** Range of individual-level community member and DPO scores for each overarching capital group (Round 5, Table 3). Mean score for each capital marked by “▲”. Note, these values correspond to the raw scores, and have not been converted to weights. **L.2)** The difference between baseline “nested” weights, and both DPO and community livelihood weights. Note, the “absolute average difference” does not account for whether the difference is positive/negative.


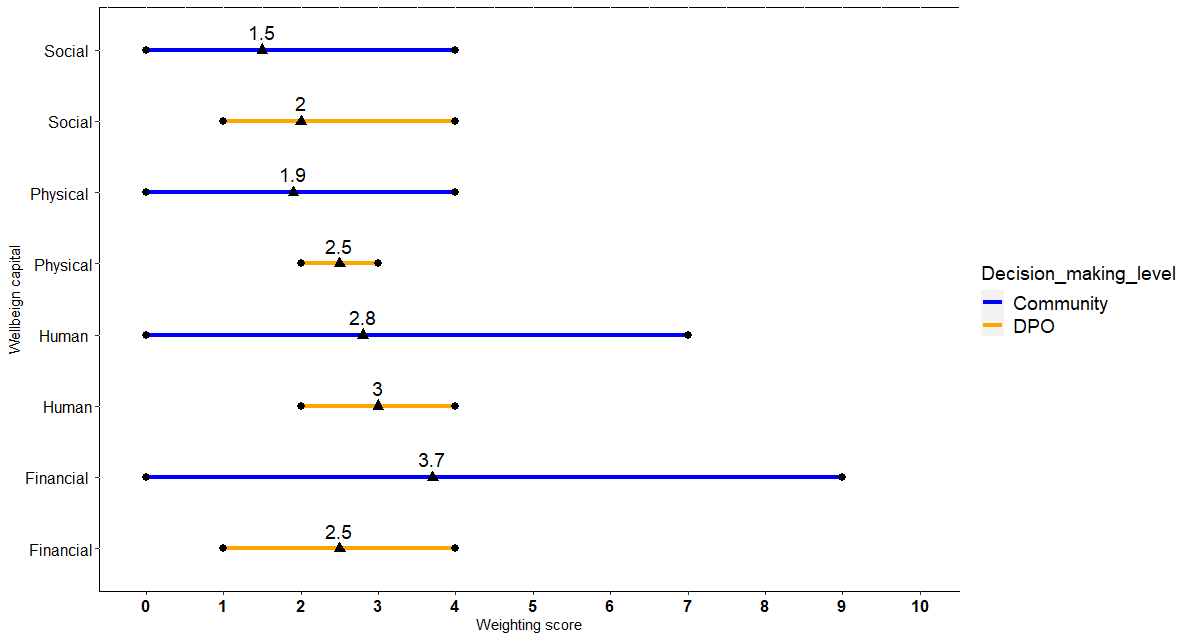


**L.1**

| **L.2** |  |  | **Difference to “nested” baseline weight** | | | |
| --- | --- | --- | --- | --- | --- | --- |
| **Capital group** | **Deprivation indicator** | **“Nested” weight** | **DPO weight** | **Farming weight** | **Fishing weight** | **Peri-urban weight** |
| **Financial** | Employment | 1.00 | +0.54 | +1.51 | +1.54 | +1.10 |
|  | Excess capital | 1.00 | +0.01 | +0.10 | +0.25 | +0.03 |
|  | Bank access | 1.00 | -0.55 | -0.02 | +0.04 | -0.44 |
| **Human** | Education | 1.50 | +0.17 | -0.12 | +0.21 | +0.22 |
|  | Healthcare access | 1.50 | +0.44 | +0.06 | +0.24 | +1.00 |
| **Social** | Cooperative | 1.50 | -0.21 | -0.70 | -0.91 | -0.94 |
|  | Network size | 1.50 | -0.39 | -0.20 | -0.53 | -0.53 |
| **Physical** | Roof | 0.60 | -0.19 | -0.36 | -0.38 | -0.41 |
|  | Drinking water | 0.60 | +0.38 | -0.04 | -0.10 | -0.09 |
|  | Toilet | 0.60 | +0.04 | -0.20 | -0.20 | -0.21 |
|  | No crowding | 0.60 | -0.26 | -0.18 | -0.33 | -0.14 |
|  | Home ownership | 0.60 | +0.04 | +0.15 | +0.18 | +0.41 |
|  | Average absolute difference to “nested” weight | | 0.27 | 0.30 | 0.41 | 0.46 |

**Appendix M** Chi-square analysis showing a significant overrepresentation of high community inequality (Gini coefficient) in highly built-up communities, and an overrepresentation of low inequality within less-urban (rural) communities.

|  | **Built-up landcover in community** | | | | |
| --- | --- | --- | --- | --- | --- |
| **Enumeration area expenditure inequality**  **(Gini coefficient)** | **None/low coverage**  **(0% - 8%)** | **Low-medium coverage**  **(9% - 19%)** | **Medium-high coverage**  **(20% - 44%)** | **High coverage (45% - 70%)** | **Total** |
| Low inequality  (0.226 – 0.320) | 233  (54%) | 87  (13%) | 57  (13%) | 51  (12%) | 428 |
| Medium inequality  (0.321 – 0.405) | 227  (43%) | 162  (31%) | 84  (16%) | 53  (10%) | 526 |
| High inequality  (0.406 – 0.536) | 192  (47%) | 79  (19%) | 53  (13%) | 86  (21%) | 410 |
| **Total** | **652**  **(48%)** | **328**  **(24%)** | **194**  **(14%)** | **190**  **(14%)** | **1,364** |
| **Chi-square test statistic** | **46.053***** | | | | |

|  | **Community basic needs deprivation rate (%)** | | | |
| --- | --- | --- | --- | --- |
| **DECCMA Enumeration area code** | **"Nested" weight** | **Community livelihood weight** | | **DPO weight** |
| 309100068 | 92 | 69 | 92 | |
| 315100055 | 90 | 69 | 79 | |
| 309100072 | 85 | 48 | 78 | |
| 405100162 (S4 – Nyitawuta) | 81 | 78 | 81 | |
| 315100071 | 79 | 71 | 86 | |
| 402200218 | 79 | 71 | 71 | |
| 405100096 | 71 | 54 | 69 | |
| 405100053 | 71 | 39 | 64 | |
| 309100006 | 69 | 69 | 65 | |
| 402200215 | 69 | 66 | 72 | |
| **315100060** | 68 | 52 | 64 | |
| 402200178 (S7 – Kedzi) | 65 | 31 | 42 | |
| **402200147** | 64 | 57 | 57 | |
| 403100024 | 64 | 46 | 64 | |
| 310100085 | 63 | 40 | 57 | |
| 309100053 | 62 | 50 | 58 | |
| 310100014 (S2 - Anyamam) | 62 | 45 | 55 | |
| 403100011 | 60 | 40 | 53 | |
| 404100161 | 59 | 38 | 62 | |
| 310100009 | 57 | 30 | 50 | |
| 310100165 | 57 | 30 | 47 | |
| 405100131 | 56 | 41 | 45 | |
| 310100024 | 55 | 48 | 52 | |
| 310100033 | 55 | 41 | 55 | |
| 404100028 | 54 | 36 | 43 | |
| 402200179 | 52 | 38 | 45 | |
| 310100145 | 50 | 40 | 47 | |
| 405100055 | 50 | 35 | 42 | |
| 402200210 | 50 | 30 | 41 | |
| 309100008 | 48 | 48 | 42 | |
| 315100086 (S1 – Afienya) | 48 | 43 | 35 | |
| 402200028 | 48 | 37 | 37 | |
| 310100005 | 47 | 43 | 47 | |
| 31010055 | 41 | 41 | 41 | |
| 404100159 (S5 – Awlikope) | 41 | 33 | 41 | |
| 404100051 | 38 | 25 | 29 | |
| 401100057 | 36 | 25 | 36 | |
| 406100094 | 29 | 29 | 36 | |
| 401100049 | 24 | 14 | 17 | |
| 402200033 (S8 - Anloga) | 21 | 24 | 17 | |
| 403100034 | 21 | 18 | 7 | |
| 406100157 | 19 | 15 | 19 | |
| 405100123 | 19 | 4 | 4 | |
| 403100076 (S6 – Aflao) | 19 | 0 | 11 | |
| 403100045 | 18 | 7 | 11 | |
| 403100058 | 17 | 7 | 14 | |
| 403100087 | 14 | 0 | 0 | |
| 406100105 | 10 | 10 | 10 | |
| 403100051 | 10 | 3 | 7 | |
| 401100139 (S3 – Sogakope) | 8 | 4 | 4 | |

**Appendix N** Community-level deprivation rates using “nested”, community livelihood and DPO weights. The ten-most deprived communities (highlighted grey for each weighting approach), using “nested” or DPO weights, excludes two communities (**highlighted blue**) which are defined within the ten-most deprived when using community livelihood weighting.
